# Assessing the current and desired levels of training and applied experiences in chronic disease prevention of students during medical school

**DOI:** 10.1186/s12909-023-04044-3

**Published:** 2023-01-23

**Authors:** Mark Stoutenberg, Lauren K. Lewis, Resa M. Jones, Francia Portacio, Denise C. Vidot, Julie Kornfeld

**Affiliations:** 1 Department of Kinesiology, College of Public Health, Temple University, 237 Pearson Hall, 1800 North Broad Street, Philadelphia, PA USA; 2 Department of Public Health, Miller School of Medicine, University of Miami, Miami, FL USA; 3 Department of Epidemiology and Biostatistics, College of Public Health, Temple University, Philadelphia, PA USA; 4 Fox Chase Cancer Center, Temple Health, Philadelphia, PA USA; 5 Combined Degree and Physician Scholars Program Office, University of Pennsylvania, Philadelphia, PA USA; 6 School of Nursing and Health Studies, University of Miami, Coral Gables, FL USA; 7 School of Public Health, Columbia University, New York, NY USA

**Keywords:** Chronic disease, Curriculum, Medical school, Physical activity, Prevention

## Abstract

**Introduction:**

Chronic diseases account for approximately 70% of deaths in the U.S. annually. Though physicians are uniquely positioned to provide behavior change counseling for chronic disease prevention, they often lack the necessary training and self-efficacy. This study examined medical student interest in receiving chronic disease prevention training as a formal part of their education as part of an effort to enhance their ability to provide guidance to patients in the future.

**Methods:**

A 23-question, online survey was sent to all undergraduate medical students enrolled in a large medical education program. The survey assessed medical student interest in receiving training related to chronic disease prevention. Survey topics included student awareness of primary prevention programs, perceived importance of receiving training and applied experience in chronic disease prevention, and preferences for how and when to receive this training.

**Results:**

Of 793 eligible medical students, 432 completed the survey (54.5%). Overall, 92.4% of students reported receiving formal training in physical activity, public health, nutrition, obesity, smoking cessation, and chronic diseases was of “very high” or “high” importance. Despite this level of importance, students most frequently reported receiving no or 1–5 h of formal training in a number of topics, including physical activity (35.4% and 47.0%, respectively) and nutrition (16.9% and 56.3%, respectively). The level of importance given to public health training was significantly greater across degree type (*p* = 0.0001) and future specialty (*p* = 0.03) for MD/MPH students and those interested in primary care, respectively.

**Conclusions:**

While medical students perceive chronic disease prevention as an important topic, most reported receiving little to no formal training. To address the growing prevalence of chronic disease across our society, programs schools should place greater emphasis on integrating training in physical activity, nutrition, and obesity-related content into the medical education curriculum.

**Supplementary Information:**

The online version contains supplementary material available at 10.1186/s12909-023-04044-3.

## Introduction

Chronic diseases, such as heart disease, cancer, and diabetes, are the leading causes of death and disability with 60% of U.S. adults having at least one chronic disease and 40% having two or more [1]. According to the National Center for Chronic Disease Prevention and Health Promotion, 90% of the nation’s $3.5 trillion in annual healthcare expenditures are for people with chronic and mental health conditions [2]. Fortunately, many chronic diseases are preventable. It is estimated that 80% of premature heart disease, stroke, and diabetes can be prevented by following a healthy diet, engaging in physical activity, moderating alcohol intake, and avoiding tobacco products [3]. Preventive care that reduces these behavioral risk factors must be considered as an integral part of primary prevention strategies to decrease the incidence of chronic diseases.

The healthcare sector has the potential to significantly impact the prevention of chronic diseases. Promotion of lifestyle behavior change via our health systems is a feasible approach to population health management as a majority of people in the U.S. (85% and 96% of adults and children, respectively) report having a usual place to receive healthcare [4]. However, physicians are inadequately prepared to offer lifestyle counseling on healthy eating, physical activity, and obesity prevention [5, 6, 7], which may be partly due to a lack of training during their medical education [8]. Multiple studies report a deficiency of training in physical activity [9] and nutrition [10] during the initial medical school experience. This trend persists as medical students advance in their training with only 14% of internal medicine interns feeling adequately trained to administer nutrition, physical activity, and obesity counseling to their future patients [11]. This trend persists through residency, with future primary care physicians reporting a lack of knowledge, self-efficacy, and proficiency to provide patients with physical activity, nutrition, and obesity counseling [8, 12, 13]. Consequently, practicing clinicians report that they are ill-prepared to provide lifestyle counseling to their patients, leading to low levels of patient counseling involving physical activity [14] and nutrition [15].

To counteract this lack of preparation, training focusing on chronic disease prevention needs to be integrated into the earliest stage of medical school and continue throughout their medical career. Undergraduate medical school is a critical first opportunity for synchronizing the needs of modern day patients with the education of future doctors [16]. Although medical schools are uniquely situated to provide instruction on engaging patients in positive health behaviors [17], only 25.0% provide dedicated training in nutrition [18] and only 56.4% of medical schools provide sufficient training in physical activity [19]. This leads to medical students who are, at most, only moderately competent in providing physical activity recommendations and obesity treatment [20, 21].

To improve the training of medical students, it is important to better understand their interests in receiving didactic and applied training experiences. This knowledge will allow better customize of their educational experience and prepare them to address the growing prevalence of chronic disease observed in our society. The purpose of this study was to assess the interests of medical students from a large medical school in receiving training in physical activity, nutrition, obesity, and chronic diseases, and applying this knowledge in real world experiences. Further, we assessed associations between medical student characteristics and the importance they report for training about physical activity, nutrition, obesity, tobacco and alcohol, public health, and chronic disease. This information may provide other medical education programs with an initial blueprint for integrating this material into their curriculum.

## Methods

### Survey development

Given that no previous work examining chronic disease training in medical school students existed at the time of this study, we designed a comprehensive 23-question, online survey to assess their interest in receiving training in topics related to chronic disease prevention. The survey consisted of questions on their demographic characteristics, lifestyle habits, awareness of primary prevention programs, perceived importance of receiving formal training and experience in chronic disease prevention, and preferences for how and when to receive this training. We assessed the content validity of the survey by sending it to several senior leaders in the medical school and made modifications based on their feedback prior to distribution (see supplemental file 1).

### Survey distribution

A link to the online survey was sent to all medical students in their first through fourth years of study at the University of Miami Leonard M. Miller School of Medicine via various student email listservs. Students received additional reminders one and two weeks after the initial email asking them to complete the survey. Prior to starting the survey, medical students were asked to read a brief introduction on the study outlining their risks and benefits to participating and provide their informed consent. All methods were carried out in accordance with relevant guidelines and regulations and all study materials and procedures were approved by the University of Miami Institutional Review Board (IRB ID#20,140,996).

### Measures and codings

The primary outcome measure of our logistic regression models was to determine the association between student characteristics and the importance of didactic training were related to physical activity, nutrition, obesity, alcohol and tobacco, and public health. Each of these variables was measured on a 5-point Likert-like scale where 1 was “very important” and 5 was “not at all important”, which were recoded as binary variables (very important/important, somewhat important/not very important/not at all important. Demographic and respondent characteristic variables included age (continuous), gender (male, female), BMI (continuous), medical school year (1–4), program (MD, MD/MPH, other), specialization (non-primary care, primary care, unsure), engages in at least 150 h of physical activity each week (yes, no), and eats at least 5 servings of fruits and/or vegetables each day (yes, no).

### Statistical analysis

At the end of the recruitment period, all responses were downloaded to an electronic, password protected file and reviewed for consistency. Estimated means were produced for individual descriptive characteristics for the overall sample, as well as by year in medical school. The prevalence of reported hours of training in topic areas, awareness of diabetes and chronic disease prevention programs, importance of receiving training within the medical education curriculum, best time for training within the curriculum, importance of training outside of the clinic setting, and best time for outside training was calculated using frequency analyses. Chi-square analyses were used to compare results by sex, year of medical school (years 1 through 4), type of medical program (MD vs. MD/MPH vs. Other), and desired medical specialty (primary care, non-primary care, unsure). Alpha levels were set at 0.05 and all tests were two-tailed. Multiple logistic regression models were run to determine the association between respondent characteristics and the self-reported importance of didactic training for: physical activity, nutrition, obesity, tobacco and alcohol, and public health. Backwards elimination with alpha < 0.15 was used for outcome-specific model building and included the following variables as potential covariates/confounders: age, gender, medical school year, program, specialization, whether they engaged in > 150 min of physical activity each week, and whether they consumed at least 5 fruits and/or vegetables each day. Analyses were performed using SAS version 9.4 (SAS Institute, Cary, NC).

## Results

In total, 432 of 793 eligible medical students (54.5%) completed the survey in an average time of seven minutes. Student demographic characteristics are presented in Table 1. The number of respondents declined across each year of medical school (1^st^ year, *n* *=* 127; 2^nd^ year, *n* = 110; 3^rd^ year, *n* = 108; 4^th^ year, *n* = 87). Slightly more than half of the respondents were female (*n* = 227, 52.5%). Across types of MD training program, 275 MD students (46.5% of all MD students), 128 MD/MPH (67.0% of all MD/MPH students), and 29 “other” MD students (i.e., MD/PhD) completed the survey. One hundred and fifty-one students (35.0%) reported being interested in entering primary care specialization, 60.0% were interested in pursuing non-primary care specialties (*n* = 259), and 5.1% were undecided on their future specialty (*n* = 22). Frequencies and percentages for the following comparisons can be found in the Supplemental Digital Content file—Additional Tables.

**Table 1:** Demographic characteristics of participating medical students

	**Overall**			**1st Year**			**2nd Year**			**3rd Year**			**4th Year**		
**Variable**	N	Mean or %	STD	N	Mean or %	STD	N	Mean or %	STD	N	Mean or %	STD	N	Mean or %	STD
Age (years)	432	25.1	2.5	127	23.7	2.12	110	25.0	2.6	108	25.8	2.3	87	26.6	2.1
Height (inches)	430	67.3	4.1	127	67.5	4.2	109	66.5	4.2	107	67.7	3.8	87	67.3	3.9
Weight (lbs)	432	149	29.7	127	150.9	28.7	110	144.8	29.8	108	151.1	30.4	87	148.9	29.9
BMI (kg/m^2^)	430	23.0	3.1	127	23.1	3.0	109	22.8	3.2	107	23.0	3.4	87	22.9	2.9
PA (min/wk)	432	167.3	133.4	127	171.2	122.5	110	163.8	127.7	108	153.4	142.1	87	183.5	144.7
FAV (serv/wk)	432	4.6	2.7	127	4.3	2.5	110	4.6	2.6	108	4.9	3	87	4.9	2.8
Gender															
Males	205	47.40%		64	50.39%		49	44.55%		53	49.07%		39	44.83%	
Females	227	52.60%		63	49.61%		61	55.45%		55	50.93%		48	55.17%	
Degree Type															
MD	275	63.70%		78	61.42%		69	62.73%		71	65.74%		57	65.52%	
MD/MPH	128	29.70%		40	31.50%		31	28.18%		29	26.85%		28	32.18%	
Other	29	6.70%		9	7.09%		10	9.09%		8	7.41%		2	2.30%	
Specialty															
Non-PC	259	59.95%		67	52.76%		64	58.18%		72	66.67%		56	64.37%	
PC	151	34.95%		48	37.80%		38	34.55%		34	31.48%		31	35.63%	
Unsure	22	5.09%		12	9.45%		8	7.27%		2	1.85%		0	0.00%	

*BMI* Body mass index, *FAV*, Self-reported consumption of fruit and vegetables, *MD*, medicine, *MPH*, Master of Public Health, *PA*, Self-reported physical activity levels, *PC*, Primary care

### Level of formal training in chronic disease-related topics

**Fig. 1:**
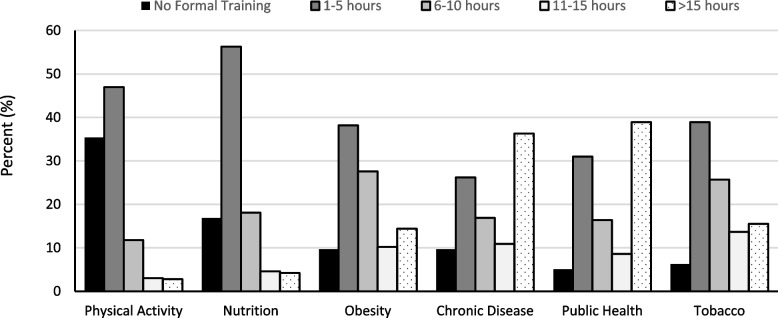
Self-reported number of hours of medical school training received in select topic areas

**Fig. 2:**
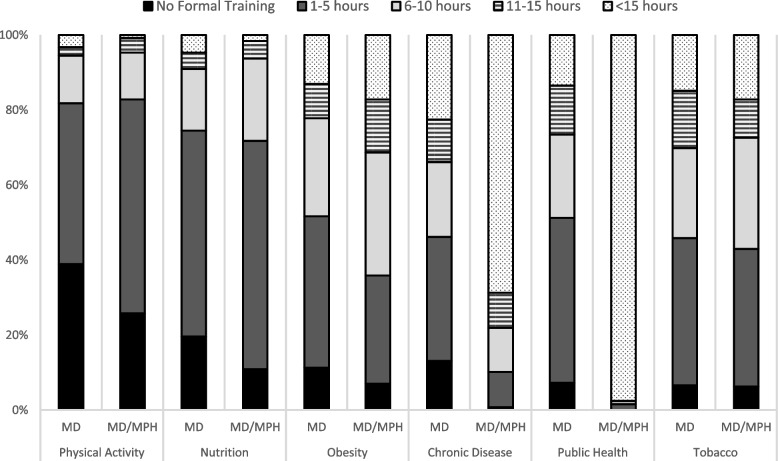
Self-reported number of hours of medical school training received in select topic areas by type of medical program

Figure 1 displays the number of self-reported hours of training that medical students received across different chronic disease-related topics. Students reported receiving less formal training (0 or 1–5 h) in physical activity (35.4% and 47.0%, respectively) and nutrition (16.9% and 56.3%, respectively), while the topics receiving the greatest amount of training (11–15 h or > 15 h) were tobacco (13.7% and 15.5%, respectively), public health (8.6% and 38.9%, respectively) and chronic diseases (10.9% and 36.3%, respectively). A significantly greater proportion of students reported receiving more training across all topics with increasing number of years in medical school. There were no differences by sex, although a trend was noted with males reporting greater levels of training on the topics of tobacco, *X*^*2*^ (4, *N* = 432) = 3.19, *p* = 0.06; and obesity, *X*^*2*^ (4, *N* = 432) = 8.33, *p* = 0.08. Significant differences were reported in the amount of training across degree programs (Fig. 2). MD students reported receiving significantly less training in physical activity, *X*^*2*^ (8, *N* = 432) = 18.89, *p* = 0.02; obesity, *X*^*2*^ (8, *N* = 432) = 15.39, *p* = 0.05; chronic disease prevention, *X*^*2*^ (8, *N* = 432) = 92.48, *p* < 0.0001; and public health, *X*^*2*^ (8, *N* = 432) = 269.15, *p* < 0.0001 than MD/MPH students. A greater proportion of students unsure of their medical specialty reported higher levels of formal training in obesity, *X*^*2*^ (8, *N* = 432) = 20.92, *p* = 0.0074); chronic disease, *X*^*2*^ (8, *N* = 432) = 18.09, *p* = 0.0206; public health, *X*^*2*^ (8, *N* = 432) = 29.14, *p* = 0.0003; and tobacco, *X*^*2*^ (8, *N* = 432) = 17.62, *p* = 0.0243.

### Awareness of major chronic disease and diabetes prevention programs

Overall, 25.7% and 47.9% of students reported being “very aware” or “somewhat aware” of major, national, or international public health programs for diabetes and chronic disease prevention, respectively. Yet only 23 students (5.3% of respondents) were able to successfully name diabetes prevention programs. Students that had completed a greater number of years in medical school were significantly more likely to report greater awareness of diabetes prevention programs, *X*^*2*^ (12, *N* = 432) = 28.01, *p* = 0.006. MD/MPH students reported having significantly greater awareness of diabetes, *X*^*2*^ (8, *N* = 432) = 65.96, *p* < 0.0001, and chronic disease, *X*^*2*^ (8, *N* = 432) = 82.37, *p* < 0.0001, prevention programs. No difference in awareness of prevention programs were observed by sex or desired medical specialty.

### Importance of formal training in topics related to chronic disease prevention

A majority of medical students felt that it was either “very important” or “important” to receive formal training on the topics of physical activity (79.0%), public health (84.0%), nutrition (90.0%), obesity (90.0%), tobacco/smoking cessation (90.1%), and chronic diseases (92.4%). No significant differences were observed in the importance of receiving formal training in these topics across year in medical school. When examined by sex, training in nutrition, *X*^*2*^ (4, *N* = 410) = 9.46, *p* = 0.05; obesity, *X*^*2*^ (4, *N* = 410) = 9.78, *p* = 0.04; and public health, *X*^*2*^ (4, *N* = 410) = 17.55, *p* = 0.002, were more important to female than male students. The level of importance given to public health training was the only topic that was significantly different across degree type, *X*^*2*^ (8, *N* = 410) = 31.47, *p* = 0.0001, and future specialty, *X*^*2*^ (8, *N* = 410) = 17.05, *p* = 0.03, with greater importance reported by MD/MPH students and those going into primary care, respectively.

### Applied experiences in primary prevention programs

**Fig. 3:**
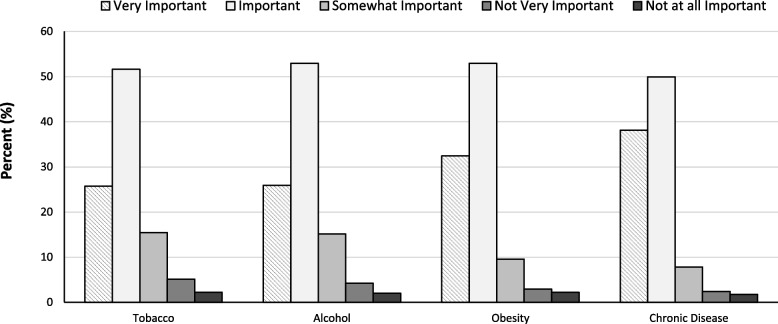
Medical student reported importance of participating in training experiences outside of the clinic setting

The importance of receiving applied experience in primary prevention programs outside of the clinical setting is displayed in Fig. 3. The majority of medical students felt it was either “important” or “very important” to have opportunities to gain experience and training outside of the classroom setting working with tobacco cessation (77.3%), alcohol misuse (78.8%), obesity (85.3%) and chronic disease (88.0%) prevention programs. Across year in medical school, the level of importance significantly differed for chronic disease prevention, *X*^*2*^ (12, *N* = 409) = 22.64, *p* = 0.03, with students earlier in their medical training reporting that this training was of greater importance. Across type of training program, a trend, *X*^*2*^ (8, *N* = 410) = 14.71, *p* = 0.07, was observed with MD/MPH students giving greater importance to receiving applied training outside of the curriculum in obesity. No significant differences in the importance of receiving applied training outside of the curriculum were observed by sex or desired medical specialty across any of the topics.

### Timing of formal training and applied experiences in obesity and primary disease prevention

When examining the best time to receive formal training in obesity and chronic disease prevention in their medical education, 48.5%, 25.6%, and 16.3% of students selected the 1^st^ through 3^rd^ years of medical school, respectively. An additional 5.1% thought this training should be included throughout their entire medical school training, while 2.9% suggested that this training be provided during residency training. There was a significant difference by year in medical school, *X*^*2*^ (15, *N* = 410) = 43.29, *p* = 0.0001, and type of degree, *X*^*2*^ (10, *N* = 410) = 21.09, *p* = 0.02, as well as a trend by sex, *X*^*2*^ (5, *N* = 410) = 9.81, *p* = 0.08). Students in their initial years of medical school, female students, and MD/MPH students all desired this training earlier in medical school. There were no significant differences observed by specialty.

### Association between respondent characteristics and the importance of prevention training

Table 2 shows the association between medical student characteristics and the self-reported importance of receiving didactic training in chronic disease-related topics. Gender was the only statistically significant independent predictor for the importance of training on physical activity, nutrition, and alcohol and tobacco with females ranging from 1.67 to 2.01 times more likely to rate receiving education in these topics as important. Student who consumed at least 5 servings of fruits and vegetables a day were 2.04 (95% CI: 1.01, 4.13) more likely to think obesity training was important than those who ate less 5 servings of fruits/vegetables per day. Finally, there were two statistically significant independent predictors of the importance of public health training. With each one-year increase in age, the odds of rating public health training as important was 0.895 (95% CI: 0.811, 0.989), while those in MD (OR = 0.18; 95% CI: 0.074, 0.441) and MD/PhD, MD/MBA, and other programs (OR = 0.18; 95% CI: 0.052, 0.626) were less likely to deem public health training important compared to those in an MD/MPH program.

**Table 2:** The association between statistically significant medical student characteristics and self-reported importance of didactic training in physical activity, nutrition, obesity, tobacco and alcohol, and public health

	**Odds Ratio**	**95% Cl**
**Physical Activity**		
Gender		
Female	1.67	1.03, 2.71
Male	Referent	
**Nutrition**		
Gender		
Female	2.01	1.03, 3.91
Male	Referent	
**Obesity**		
Eats 5 + fruits/vegetables per day		
Yes	2.04	1.01, 4.13
No	Referent	
Gender		
Female	2.01	1.03, 3.91
Male	Referent	
**Public Health**		
Age	0.895	0.811, 0.989*
Gender		
Female	1.68	0.958, 2.94
Male	Referent	
Program Type		
MD	0.181	0.074, 0.441
MD/PhD, MD/MBA, other	0.181	0.052, 0.626
MD/MPH	Referent	
Exercises > 150 min per week		
Yes	0.601	0.345, 1.05
No	Referent	

## Discussion

With chronic diseases being the leading cause of death and disability in the U.S., accounting for more than $3.5 trillion in annual health care costs [22], it is imperative to optimize the training of our future health care workforce. Medical schools play a vital role enhancing primary prevention training and preparing physicians to reduce preventable deaths caused by chronic diseases. Physicians are in a unique position to influence their patients; thus, it is essential that they feel competent in providing recommendations for engagement in healthy lifestyle behaviors. This study assessed medical student perceptions on receiving training in physical activity, nutrition, obesity, and chronic diseases, and their preferences for applying this knowledge in applied activities outside of the classroom setting.

Several key findings emerged from this study, summarized in Table 3, along with potential future actions that might be considered by medical schools. Nearly all (92.4%) medical students at this institution attached a high level of importance to receiving chronic disease training during medical school, a level exceeding the desires of medical students (76%) in the United Kingdom for incorporation of lifestyle medicine and physical activity teaching into their curriculum [23]. Students in our study also desired greater exposure to primary prevention programs as a part of their medical training. However, only a quarter of students reported being “very aware” of public health prevention programs and, when prompted, only 5% correctly listed an evidence-based chronic disease program (i.e., the diabetes prevention program). This lack of awareness was similarly observed in a previous study in which medical students correctly answered fewer than half of the questions related to prediabetes and diabetes prevention [24]. This lack of knowledge suggests either an absence of exposure to chronic disease prevention programs or an inability to retain information covered during their medical school training.

**Table 3:** Summary of findings: Medical student perceptions on receiving training in physical activity, nutrition, obesity, and chronic diseases

Topic	Findings	Potential Actions
Importance of receiving formal chronic disease prevention training	• Nearly all medical students felt it was important to receive chronic disease prevention training during medical school	• Consider public health as a core discipline in higher education to help students view medicine through a population health lens • Place greater emphasis on primary and secondary disease prevention throughout all stages of medical education
Level of training in topics related to chronic disease prevention	• Medical students most frequently reported receiving 0 or 1–5 h of formal training in physical activity and nutrition	• Consider physical activity, nutrition, and obesity as integral components of the medical education curriculum • Assess knowledge in chronic disease prevention via board and certification exams
Awareness of chronic disease prevention programs	• A small percentage of medical students were aware of and can correctly list chronic disease prevention programs	• Increase exposure to evidence-based programs (e.g., the CDC Community Guide) and offer more real-world experiences related to chronic disease prevention
Individuals desiring training in chronic disease prevention	• Medical students in their initial years of medical school, female students, and MD/MPH students place greater importance on receiving training in chronic disease prevention	• Increase exposure and broaden the appeal of chronic disease prevention to all students, especially male students and those entering non-primary care fields • Utilize more simulated patients and case studies in empathy training to address gender-empathy gap
Best time to receive training in chronic disease prevention	• The initial years of medical school were identified as the best time to receive training in chronic disease prevention, especially for female students, MD/MPH students, and students in their initial years of medical school	• Introduce students to chronic disease prevention earlier in their medical education • Continue emphasizing chronic disease prevention throughout all years of medical school
Importance of receiving applied experience	• Most medical students feel receiving applied experiences is important and desire exposure to real-world applications of chronic disease prevention programs	• Consider applied experiences related to chronic disease prevention such as: ◦ setting personal goals to modify their own lifestyle behaviors ◦ self-monitoring of their lifestyle habits ◦ field trips (i.e., grocery stores, community cooking classes)

In addition to didactic training, medical students desired greater exposure to the real-world application of chronic disease programs. Hivert et al [7] suggested that lifestyle-related knowledge and skill development should incorporate a combination of didactic and experiential learning opportunities, including observational and applied work with peers in clinical settings. Examples of applied activities include having students set personal goals to modify their own lifestyle behaviors to experience the behavior change process, self-monitoring of their lifestyle habits (e.g., dietary intake, physical activity, screen time, sleep pattern, alcohol intake), and participating in educational field trips (e.g., grocery stores, cooking classes). However, such activities require additional time, resources, and creative planning for successful implementation and are not a common part of medical school curricula.

We also noted a higher level of student interest in public health and receiving training on chronic disease prevention earlier in the medical education program, particularly as reported by students earlier in their training program. One explanation for this may be the idealism of the profession when students first start, which gradually changes as they observe the reality of medicine [25]. The decline in idealism has been detected as early as the second year of medical training with student motivation often impacted by student debt over interest in content or specialty choice [26]. Alternatively, students further into their medical training may consider public health-related training to be less important due to the influence of their experiences in clinical settings. Given the importance of disease prevention, medical students may benefit from a more intensive introduction to prevention earlier in their medical education. Increasing awareness of the meaningful work of primary care fields and improving the delivery of self-care practices to decrease the burnout rate associated with primary care specialties may also more positively shape the physician workforce [27, 28].

Our findings also reflect gender differences with female students being 67–101% more likely to rate receiving training in chronic disease prevention topics as important. Gender was the only statistically significant independent predictor for the importance of training on physical activity, nutrition, and alcohol and tobacco with females ranging from 1.67 to 2.01 times more likely to rate receiving education in these topics as important. Female providers tend to be more receptive to emotional signals than males and therefore achieve a more empathetic relationship with their patients [29, 30], serving as a valuable ally to the care of patients in preventing, diagnosing, and managing chronic diseases [31, 32]. Studies suggest that students who prefer people-oriented specialties have higher empathy scores compared to those who prefer technology and procedure-oriented specialties [33, 34]. Applied experiences, such as utilizing standardized patients as part of an educational empathy training program [35], may be a helpful addition to closing this gender-empathy gap.

The MD/MPH students in our study gave greater importance to training in public health, physical activity, obesity, and chronic disease prevention compared to their traditional MD counterparts. The University of Miami Miller School of Medicine offers a fully integrated MD/MPH program that provides innovative training that combines clinical medicine and public health. Medical students receive in-depth training in both public health and medicine, providing them with the skills to reduce death and disability at a population level. Future work may wish to explore the extent to which students with a stronger interest in physical activity, obesity and chronic disease prevention were drawn to a combined MD/MPH program versus developing this interest through the public health-related topics integrated into their studies. Additionally, previous work has demonstrated that MD/MPH students are more inclined to seek people-oriented specialties, such as family medicine, that have a greater focus on preventive activities [36]. This highlights the importance of integrating public health into medical education earlier in programs wishing to increase their focus on preventive care. Ultimately, public health should be considered a core discipline in higher education to increase student empathy and allow students to view medicine with a greater public health mindset [37].

To apply these findings into practice, medical schools must overcome several barriers. One set of important barriers is the time and resource constraints placed on medical institutions. Due to numerous existing competencies, medical schools often lack flexibility to include additional content in an already packed curriculum [38, 39, 40]. The Crimson Care Collaborative at Harvard Medical School, a student-faculty collaborative practice, overcomes this barrier by offering evidence-based education and training sessions focused on chronic disease management, exploring patient priorities, providing focused counseling and education, and assisting patients with self-management goals during clinical visits [41]. Another example is the Profession MD–Lifestyle Program implemented by the University of Sherbrooke. This longitudinal program provides students with the knowledge and skills to support healthy lifestyle behavior, while aligning sessions with the system-based thematic modules, integrated complex medical problem sets, and practical clinical vignettes with standardized patients in the last preclinical semester [42].

To implement new training models, institutions must adopt educational goals beyond the traditional medical education curriculum and have champions drive the initiative forwards. The University of South Carolina School of Medicine Greenville designed, developed, and implemented an innovative, formalized lifestyle medicine curriculum based on chronic disease prevention as a core foundation of their undergraduate medical student training [43]. The adoption of a comprehensive lifestyle medicine curriculum as an integral part of the medical school’s culture is largely attributed to buy-in at the Dean’s level and key faculty who championed efforts to coordinate its integration across the entire curriculum [43, 44].

There are several strengths to this study. The survey was easily administered, resulting in a high response rate (54.5% of all eligible medical students) that represents a cross-sectional sample of the medical school student population at the institution (i.e., year in school, sex, age) [45]. However, this study has several limitations. One limitation is a lack of external generalizability as perceptions and experiences came from students at only one institution. Participation in the study was voluntary, anonymous, and open to selection bias as more students with positive opinions towards chronic disease prevention training may have responded. Additionally, some key terms, such as ‘public health’, were not explicitly defined. Further, the sample is overrepresented by MD/MPH students compared to MD students (67.0% versus 46.5% response rate, respectively), who may have been drawn to this innovative program. Despite these limitations, this study raises awareness of important gaps in providing primary prevention training in a medical school training program, provides a framework to repeat similar formative work on a larger scale with other institutions, and offers a starting point for implementing this training in medical school curriculums.

## Conclusions

This study is one of the first to investigate medical student interest in receiving didactic and applied training experiences in chronic disease prevention. While the majority of medical students considered training in chronic disease prevention to be important, there was an apparent lack of knowledge and overall training. Medical school curricula should include an array of diverse experiences to provide physical activity, nutrition, and chronic disease prevention content that is emphasized throughout the curriculum and through real-world experiences. We hope that the findings from this study start further conversations that lead to a greater emphasis on the integration of chronic disease prevention training and applied experiences into medical school curricula.

## Supplementary Information

**Additional file 1.** Medical Student Survey.

**Additional file 2:** **Supplemental Table 1. **Hours of formal training in chronic disease-related topics, overall. **Supplemental Table 2. **Hours of formal training in chronic disease prevention related-topics by year in medical school. **Supplemental Table 3.** Hours of formal training in chronic disease prevention related-topics by sex. **Supplemental Table 4.** Hours of formal training in chronic disease prevention related-topics by degree type. **Supplemental Table 5.** Hours of formal training in chronic disease prevention related-topics by medical specialty. **Supplemental Table 6.** Awareness of major chronic disease and diabetes prevention programs, overall. **Supplemental Table 7.** Awareness of major chronic disease and diabetes prevention programs by year in medical school. **Supplemental Table 8.** Awareness of major chronic disease and diabetes prevention programs by sex. **Supplemental Table 9.** Awareness of major chronic disease and diabetes prevention programs by degree type. **Supplemental Table 10.** Awareness of major chronic disease and diabetes prevention programs by medical specialty. **Supplemental Table 11.** Importance of formal training in chronic disease-related topics, overall. **Supplemental Table 12.** Importance of formal training in chronic disease-related topics by year in medical school. **Supplemental Table 13.** Importance of formal training in chronic disease-related topics by sex. **Supplemental Table 14.** Importance of formal training in chronic disease-related topics by degree type. **Supplemental Table 15.** Importance of formal training in chronic disease-related topics by medical specialty. **Supplemental Table 16.** Importance of applied experiences in chronic disease prevention, overall. **Supplemental Table 17.** Importance of applied experiences in chronic disease prevention by year in medical school. **Supplemental Table 18.** Importance of applied experiences in chronic disease prevention by sex. **Supplemental Table 19.** Importance of applied experiences in chronic disease prevention by degree type. **Supplemental Table 20.** Importance of applied experiences in chronic disease prevention by medical specialty. **Supplemental Table 21.** Best time to receive formal training in chronic disease prevention, overall. **Supplemental Table 22.** Best time to receive formal training in chronic disease prevention by year in medical school. **Supplemental Table 23.** Best time to receive formal training in chronic disease prevention by sex. **Supplemental Table 24**. Best time to receive formal training in chronic disease prevention by degree type. **Supplemental Table 25.** Best time to receive formal training in chronic disease prevention by medical specialty. **Supplemental Table 26.** Best time to receive applied experiences in chronic disease prevention, overall. **Supplemental Table 27.** Best time to receive applied experiences in chronic disease prevention by year in medical school. **Supplemental Table 28.** Best time to receive applied experiences in chronic disease prevention by sex. **Supplemental Table 29.** Best time to receive applied experiences in chronic disease prevention by degree type. **Supplemental Table 30.** Best time to receive applied experiences in chronic disease prevention by medical specialty.

## Data Availability

The dataset generated and analyzed during the current study is publicly available in Figshare, a data repository (https://doi.org/10.6084/m9.figshare.21626534.v1).

## Abbreviations

MD: Medicine
MPH: Master of Public Health
U.S.: United States

## References

[1] U.S. Center for Disease Control and Prevention. Chronic Diseases in America. https://www.cdc.gov/chronicdisease/resources/infographic/chronic-diseases.htm. Accessed: 19 Nov 2022.

[2] U.S. Center for Disease Control and Prevention. Health and Economic Costs of Chronic Diseases. https://www.cdc.gov/chronicdisease/about/costs/index.htm. Accessed: 23 Mar 2020.

[3] Overview - Preventing Chronic Diseases: A Vital Investment. https://www.who.int/chp/chronic_disease_report/part1/en/index11.html. Accessed: 11 Apr 2020.

[4] Ashman JJ, Santo L, Okeyode T. Characteristics of office-based physician visits, 2018 NCHS Data Brief, no 408 Hyattsville MD. National Center for Health Statistics. 2021. 10.15620/cdc:105509. 33983876

[5] Nair D, Hart A (2018). Family physicians' perspectives on their weight loss nutrition counseling in a high obesity prevalence area. J Am Board Fam Med.

[6] Crowley J, Ball L, Hiddink GJ (2019). Nutrition in medical education: A systematic review. Lancet Planetary Healt.

[7] Hivert MF, Arena R, Forman DE (2016). Medical training to achieve competency in lifestyle counseling: an essential foundation for prevention and treatment of cardiovascular diseases and other chronic medical conditions: A scientific statement from the American Heart Association. Circulation.

[8] Smith S, Seeholzer EL, Gullett H (2015). Primary care residents' knowledge, attitudes, self-efficacy, and perceived professional norms regarding obesity, nutrition, and physical activity counseling. J Grad Med Educ.

[9] Brannan M, Bernardotto M, Clarke N, Varney J (2019). Moving healthcare professionals – a whole system approach to embed physical activity in clinical practice. BMC Med Educ.

[10] Adams KM, Butsch WS, Kohlmeier M (2015). The state of nutrition education at US medical schools. J Biomed Educ.

[11] Vetter ML, Herring SJ, Sood M, Shah NR, Kalet AL (2008). What do resident physicians know about nutrition? An evaluation of attitudes, self-perceived proficiency and knowledge. J Am Coll Nutr.

[12] Antognoli EL, Seeholzer EL, Gullett H, Jackson B, Smith S, Flocke SA (2017). Primary care resident training for obesity, nutrition, and physical activity counseling: A mixed-methods study. Health Promot Pract.

[13] Devries S, Dalen JE, Eisenberg DM (2014). A deficiency of nutrition education in medical training. Am J Med.

[14] Ahmed NU, Delgado M, Saxena A (2017). Trends and disparities in the prevalence of physicians' counseling on exercise among the U.S. adult population, 2000–2010. Prev Med..

[15] Kris-Etherton PM, Akabas SR, Bales CW (2014). The need to advance nutrition education in the training of health care professionals and recommended research to evaluate implementation and effectiveness. Am J Clin Nutr.

[16] Colbert JA, Jangi S (2013). Training physicians to manage obesity - back to the drawing board. N Engl J Med.

[17] Vijn TW, Fluit C, Kremer JAM, Beune T, Faber MJ, Wollersheim H (2017). Involving medical students in providing patient education for real patients: A scoping review. J Gen Intern Med.

[18] Adams KM, Kohlmeier M, Zeisel SH (2010). Nutrition education in U.S. medical schools Latest update of a national survey. Acad Med..

[19] Stoutenberg M, Stasi S, Stamatakis E (2015). Physical activity training in US medical schools: Preparing future physicians to engage in primary prevention. Phys Sportsmed.

[20] Butsch WS, Kushner RF, Alford S, Smolarz BG (2020). Low priority of obesity education leads to lack of medical students' preparedness to effectively treat patients with obesity: Results from the U.S. medical school obesity education curriculum benchmark study. BMC Med Educ..

[21] Vitolins MZ, Crandall S, Miller D, Ip E, Marion G, Spangler JG (2012). Obesity educational interventions in U.S. medical schools: A systematic review and identified gaps. Teach Learn Med..

[22] National Center for Chronic Disease Prevention and Health Promotion: About Chronic Diseases. https://www.cdc.gov/chronicdisease/about/index.htm. Accessed: 8 Jul 2020.

[23] Radenkovic D, Aswani R, Ahmad I, Robinson R, Kreindler (2019). Lifestyle medicine and physical activity knowledge of final year UK medical students. BMJ Open Sport Exerc Med..

[24] Khan T, Wozniak GD, Kirley K (2019). An assessment of medical students' knowledge of prediabetes and diabetes prevention. BMC Med Educ.

[25] Sethia B (2013). In praise of idealism in healthcare. J R Soc Med.

[26] Mader EM, Roseamelia C, Morley CP (2014). The temporal decline of idealism in two cohorts of medical students at one institution. BMC Med Educ.

[27] Shanafelt TD, Boone S, Tan L (2012). Burnout and satisfaction with work-life balance among US physicians relative to the general US population. Arch Intern Med.

[28] Clinite KL, DeZee KJ, Durning SJ (2014). Lifestyle factors and primary care specialty selection: Comparing 2012–2013 graduating and matriculating medical students' thoughts on specialty lifestyle. Acad Med.

[29] Bertakis KD, Helms LJ, Callahan EJ, Azari R, Robbins JA (1995). The influence of gender on physician practice style. Med Care.

[30] Howick J, Steinkopf L, Ulyte A, Roberts N, Meissner K (2017). How empathic is your healthcare practitioner? A systematic review and meta-analysis of patient surveys. BMC Med Educ.

[31] Kourakos M, Vlachou E, Kelesi M (2018). Empathy in the health professions: An ally in the care of patients with chronic diseases. Int J Med Health Sci.

[32] Beale L (2017). The emotional life of patients with chronic diseases: A framework for health promotion strategies. Int J Med Health Sci.

[33] Hojat M, Vergare MJ, Maxwell K (2009). The devil is in the third year: A longitudinal study of erosion of empathy in medical school. Acad Med.

[34] Chen DC, Kirshenbaum DS, Yan J, Kirshenbaum E, Aseltine RH (2012). Characterizing changes in student empathy throughout medical school. Med Teach.

[35] Ferri P, Rovesti S, Padula MS, D'Amico R, Di Lorenzo R (2019). Effect of expert-patient teaching on empathy in nursing students: A randomized controlled trial. Psychol Res Behav Manag.

[36] Andriole DA, Jeffe DB, Tai RH (2016). Characteristics and career intentions of MD-MPH program graduates: A national cohort study. Public Health Rep.

[37] Maddock JE, Moore JB (2020). Should public health literacy be a core requirement for college students?. J Public Health Manag Pract.

[38] Kutaimy R, Zhang L, Blok D (2018). Integrating patient safety education into early medical education utilizing cadaver, sponges, and an inter-professional team. BMC Med Educ.

[39] Gonzalo JD, Caverzagie KJ, Hawkins RE, Lawson L, Wolpaw DR, Chang A (2018). Concerns and responses for integrating health systems science into medical education. Acad Med.

[40] van Schaik SM, Reeves SA, Headrick LA (2019). Exemplary learning environments for the health professions: A vision. Acad Med.

[41] Bodenheimer T, Handley MA (2009). Goal-setting for behavior change in primary care: An exploration and status report. Patient Educ Couns.

[42] Pate R, Buchner D (2014). Implementing Physical Activity.

[43] Trilk JL, Elkhider IA, Asif I (2019). Design and implementation of a lifestyle medicine curriculum in undergraduate medical education. Am J Lifestyle Med.

[44] Muscato D, Phillips EM, Trilk JL (2018). Lifestyle medicine education collaborative (LMEd): "Champions of Change" medical school leaders workshop. Am J Lifestyle Med.

[45] Association of American Medical Colleges. Applicants, first-time applicants, accepted, and matriculants to U.S. medical schools by sex, 2010–2011 through 2019–2020. https://www.aamc.org/system/files/2019-10/2019_FACTS_Table_A-7.2.pdf. Accessed: 8 Jul 2020.

