# Supplementary material for: Assessing the current and desired levels of training and applied experiences in chronic disease prevention of students during medical school
Source: BMC Med Educ. 2023 Jan 23;23:54. doi: 10.1186/s12909-023-04044-3 (PMC9872306; doi:10.1186/s12909-023-04044-3)
Supplement: Supplementary file 1 — Additional file 1. Medical Student Survey. [file 12909_2023_4044_MOESM1_ESM.docx]

**Assessing the current and desired levels of training and
applied experiences in chronic diseases during medical school**

1. What is your age?
2. What is your gender?

- Male
- Female

1. What is your current year of medical education?

- 1st Year
- 2nd Year
- 3rd Year
- 4th Year

1. Which degree program are you currently enrolled in?

- MD
- MD/MPH
- MD/PhD
- MD/MBA
- Others - which? ____________________

1. What is your desired area of medical specialization? Options:

- Surgical (ENT, Ortho, etc.)
- Primary Care (Internal Medicine, Pediatrics, Family Medicine)
- Psychiatry
- Obstetrics & Gynecology
- Other (please indicate which): ____________________

1. What is your current height (in inches)?
2. What is your current weight (in pounds)?
3. Do you currently smoke?

- Yes
- No

If No is selected, then skip to end of block

8a. Do you smoke:

- Regularly
- Occasionally
- Socially

1. In a typical week, how many days a week do you engage in moderate (i.e., brisk walking) to vigorous (i.e., running, strength training, fast cycling) physical activity?

- No days
- One day
- Two days
- Three days
- Four days
- Five days
- Six days
- Seven days

1. On a normal day, for how many minutes do you engage in this physical activity?
2. How many servings of fruit do you consume on a typical day?
3. How many servings of vegetables do you consume on a typical day?
4. How many hours of formal education (i.e., in class training) have you received during your time in medical school in each of the following:

|  | 1-5 hours | 6-10 hours | 11-15 hours | >15 hours | No formal training |
| --- | --- | --- | --- | --- | --- |
| Physical Activity |  |  |  |  |  |
| Nutrition |  |  |  |  |  |
| Obesity |  |  |  |  |  |
| Tobacco and Alcohol |  |  |  |  |  |
| Public Health |  |  |  |  |  |
| Health Promotion |  |  |  |  |  |
| Chronic Disease Prevention (e.g., diabetes prevention programs) |  |  |  |  |  |

1. How would you assess your awareness of major public health programs developed for primary prevention of chronic diseases?

- Very Aware
- Somewhat Aware
- Neutral
- Somewhat Unaware
- Not at all Aware

1. Please mention examples of some of the programs that you are aware of:
2. How would you assess your awareness of major national or international diabetes prevention programs?

- Very Aware
- Somewhat Aware
- Neutral
- Somewhat Unaware
- Not at all Aware

1. Please mention examples of diabetes prevention programs that you are aware of.
2. How important do you consider it to be that you receive formal training in the following during your medical education:

|  | Very important | | Important | | Somewhat Important | | Not Very Important | | Not at all Important | |  |
| --- | --- | --- | --- | --- | --- | --- | --- | --- | --- | --- | --- |
| Physical Activity | |  | |  | |  | |  | |  | |
| Nutrition | |  | |  | |  | |  | |  | |
| Obesity | |  | |  | |  | |  | |  | |
| Tobacco and Alcohol | |  | |  | |  | |  | |  | |
| Public Health | |  | |  | |  | |  | |  | |
| Health Promotion | |  | |  | |  | |  | |  | |
| Chronic Disease Prevention (i.e., prevention programs) | |  | |  | |  | |  | |  | |

1. In your opinion, when would be the best time for you to receive formal training in obesity (i.e., physical activity, nutrition) and primary disease prevention?

- During the 1st Year of Medical School
- During the 2nd Year of Medical School
- During the 3rd Year of Medical School
- During my Residency
- During my Internship/Fellowship
- Never
- Other (please list): ____________________

If Never is selected, then skip to: Q22.

1. How would you like to receive this training? Please check all that apply.

- Required classes and seminars
- Elective classes and seminars
- Clinical experiences
- Community outreach programs (i.e., student health fairs)
- Working directly with prevention programs in the community
- Other (please indicate): ____________________

1. How important is it to you to receive applied experiences (i.e., directly working with these populations of individuals OUTSIDE of a clinical setting) in the following areas during your medical education:

|  | Very Important | Important | Not Important | Not Very Important | Not at all Important |
| --- | --- | --- | --- | --- | --- |
| Smoking cessation programs |  |  |  |  |  |
| Alcohol treatment programs |  |  |  |  |  |
| Obesity prevention & intervention programs |  |  |  |  |  |
| Chronic disease prevention programs |  |  |  |  |  |

1. When would be the best time for you to receive applied experience (i.e., participating in the delivery of prevention or management programs) in obesity and primary disease prevention?

- During the 1st Year of Medical School
- During the 2nd Year of Medical School
- During the 3rd Year of Medical School
- During the 4th Year of Medical School
- During my residency
- During my fellowship/internship
- Another time (please indicate): ____________________
