# Supplementary material for: Assessing the current and desired levels of training and applied experiences in chronic disease prevention of students during medical school
Source: BMC Med Educ. 2023 Jan 23;23:54. doi: 10.1186/s12909-023-04044-3 (PMC9872306; doi:10.1186/s12909-023-04044-3)
Supplement: Supplementary file 2 — Additional file 2: Supplemental Table 1. Hours of formal training in chronic disease-related topics, overall. Supplemental Table 2. Hours of formal training in chronic disease prevention related-topics by year in medical school. Supplemental Table 3. Hours of formal training in chronic disease prevention related-topics by sex. Supplemental Table 4. Hours of formal training in chronic disease prevention related-topics by degree type. Supplemental Table 5. Hours of formal training in chronic disease prevention related-topics by medical specialty. Supplemental Table 6. Awareness of major chronic disease and diabetes prevention programs, overall. Supplemental Table 7. Awareness of major chronic disease and diabetes prevention programs by year in medical school. Supplemental Table 8. Awareness of major chronic disease and diabetes prevention programs by sex. Supplemental Table 9. Awareness of major chronic disease and diabetes prevention programs by degree type. Supplemental Table 10. Awareness of major chronic disease and diabetes prevention programs by medical specialty. Supplemental Table 11. Importance of formal training in chronic disease-related topics, overall. Supplemental Table 12. Importance of formal training in chronic disease-related topics by year in medical school. Supplemental Table 13. Importance of formal training in chronic disease-related topics by sex. Supplemental Table 14. Importance of formal training in chronic disease-related topics by degree type. Supplemental Table 15. Importance of formal training in chronic disease-related topics by medical specialty. Supplemental Table 16. Importance of applied experiences in chronic disease prevention, overall. Supplemental Table 17. Importance of applied experiences in chronic disease prevention by year in medical school. Supplemental Table 18. Importance of applied experiences in chronic disease prevention by sex. Supplemental Table 19. Importance of applied experiences in chronic disease prevention by d [file 12909_2023_4044_MOESM2_ESM.docx]

**Supplemental Table 1. Hours of formal training in chronic disease-related topics, overall.**

|  | No Formal Training | | 1-5 hours | | 6-10 hours | | 11-15 hours | | >15 hours | |
| --- | --- | --- | --- | --- | --- | --- | --- | --- | --- | --- |
|  | Frequency | Percent | Frequency | Percent | Frequency | Percent | Frequency | Percent | Frequency | Percent |
| Physical Activity | 153 | 35.4 | 203 | 47.0 | 51 | 11.8 | 13 | 3 | 12 | 2.8 |
| Nutrition | 73 | 16.9 | 243 | 56.3 | 78 | 18.1 | 20 | 4.6 | 18 | 4.2 |
| Obesity | 42 | 9.7 | 165 | 38.2 | 119 | 27.6 | 44 | 10.2 | 62 | 14.4 |
| Chronic Disease | 42 | 9.7 | 113 | 26.2 | 73 | 16.9 | 47 | 10.9 | 157 | 36.3 |
| Public Health | 22 | 5.1 | 134 | 31 | 71 | 16.4 | 37 | 8.6 | 168 | 38.9 |
| Tobacco | 27 | 6.3 | 168 | 38.9 | 111 | 25.7 | 59 | 13.7 | 67 | 15.5 |

**Supplemental Table 2. Hours of formal training in chronic disease prevention related-topics by year in medical school.**

|  | No Formal Training | | 1-5 hours | | 6-10 hours | | 11-15 hours | | >15 hours | | Chi-Square Test Results |
| --- | --- | --- | --- | --- | --- | --- | --- | --- | --- | --- | --- |
|  | Frequency | Percent | Frequency | Percent | Frequency | Percent | Frequency | Percent | Frequency | Percent |  |
| **Physical Activity** |  | |  |  |  |  |  |  |  |  |  |
| 1st year | 55 | 43.3 | 57 | 44.9 | 10 | 7.9 | 3 | 2.4 | 2 | 1.6 | df = 12 |
| 2nd year | 42 | 38.2 | 56 | 50.9 | 8 | 7.3 | 0 | 0 | 4 | 3.6 | *x^2^* = 22.66 |
| 3rd year | 27 | 25 | 52 | 48.2 | 20 | 18.5 | 6 | 5.6 | 3 | 2.8 | p=0.0307 |
| 4th year | 29 | 33.3 | 38 | 43.7 | 13 | 14.9 | 4 | 4.6 | 3 | 3.5 |  |
| **Nutrition** |  | |  | |  | |  | |  | |  |
| 1st year | 50 | 39.4 | 60 | 47.2 | 13 | *x^2^* = | 3 | 2.4 | 1 | 1 | df = 12 |
| 2nd year | 6 | 5.5 | 76 | 69.1 | 20 | 18.2 | 3 | 2.7 | 5 | 4.6 | *x^2^* = 78.34 |
| 3rd year | 9 | 8.3 | 60 | 55.6 | 26 | 24.1 | 6 | 5.6 | 7 | 6.5 | p<.0001 |
| 4th year | 8 | 9.2 | 47 | 54 | 19 | 21.8 | 8 | 9.2 | 5 | 5.8 |  |
| **Obesity** |  | |  | |  | |  | |  | |  |
| 1st year | 35 | 27.6 | 57 | 44.9 | 21 | 16.5 | 11 | 8.7 | 3 | 2.4 | df = 12 |
| 2nd year | 3 | 2.7 | 55 | 50 | 32 | 29.1 | 7 | 6.4 | 13 | 11.8 | *x^2^* = 110.75 |
| 3rd year | 3 | 2.8 | 25 | 23.2 | 41 | 38 | 13 | 12 | 26 | 24.1 | p<.0001 |
| 4th year | 1 | 1.2 | 28 | 32.2 | 25 | 28.7 | 13 | 14.9 | 20 | 23 |  |
| **Chronic Disease** |  | |  | |  | |  | |  | |  |
| 1st year | 29 | 22.8 | 39 | 30.7 | 19 | 15 | 11 | 8.7 | 29 | 22.8 | df = 12 |
| 2nd year | 4 | 3.6 | 35 | 31.8 | 19 | 17.3 | 11 | 10 | 41 | 37.27 | *x^2^* = 49.77 |
| 3rd year | 5 | 4.6 | 23 | 21.3 | 21 | 19.4 | 13 | 12 | 46 | 42.6 | p<.0001 |
| 4th year | 4 | 4.6 | 16 | 18.4 | 14 | 16.1 | 12 | 13.8 | 41 | 47.1 |  |
| **Public Health** |  | |  | |  | |  | |  | |  |
| 1st year | 17 | 13.4 | 53 | 41.7 | 14 | 11 | 3 | 2.4 | 40 | 31.5 | df = 12 |
| 2nd year | 0 | 0 | 30 | 27.3 | 25 | 22.7 | 11 | 10 | 44 | 40 | *x^2^* = 50.20 |
| 3rd year | 1 | 0.9 | 31 | 28.7 | 17 | 15.7 | 14 | 13 | 45 | 41.7 | p<.0001 |
| 4th year | 4 | 4.6 | 20 | 23 | 15 | 17.2 | 9 | 10.3 | 39 | 44.8 |  |
| **Tobacco** |  |  |  |  |  |  |  |  |  |  |  |
| 1st year | 21 | 16.5 | 70 | 55.1 | 26 | 20.5 | 5 | 3.9 | 5 | 3.9 | df = 12 |
| 2nd year | 2 | 1.8 | 47 | 42.7 | 33 | 30 | 16 | 14.6 | 12 | 10.9 | *x^2^* = 94.54 |
| 3rd year | 1 | 0.9 | 29 | 26.9 | 34 | 31.5 | 21 | 19.4 | 23 | 21.3 | p<.0001 |
| 4th year | 3 | 3.5 | 22 | 25.3 | 18 | 20.7 | 17 | 19.5 | 27 | 31 |  |

**Supplemental Table 3. Hours of formal training in chronic disease prevention related-topics by sex.**

|  | No Formal Training | | 1-5 hours | | 6-10 hours | | 11-15 hours | | >15 hours | | Chi-Square Test Results |
| --- | --- | --- | --- | --- | --- | --- | --- | --- | --- | --- | --- |
|  | Frequency | Percent | Frequency | Percent | Frequency | Percent | Frequency | Percent | Frequency | Percent |  |
| **Physical Activity** |  |  |  |  |  |  |  |  |  |  | df = 4 |
| Males | 73 | 35.6 | 90 | 43.9 | 29 | 14.2 | 6 | 2.9 | 7 | 3.4 | *x^2^* = 3.19 |
| Females | 80 | 35.2 | 113 | 49.8 | 22 | 9.7 | 7 | 3.1 | 5 | 2.2 | p=0.5273 |
| **Nutrition** |  |  |  |  |  |  |  |  |  |  | df = 4 |
| Males | 39 | 19 | 102 | 49.8 | 43 | 21 | 11 | 5.4 | 10 | 4.9 | *x^2^* = 6.74 |
| Females | 34 | 15 | 141 | 62.1 | 35 | 15.4 | 9 | 4 | 8 | 3.5 | p=0.1502 |
| **Obesity** |  |  |  |  |  |  |  |  |  |  | df = 4 |
| Males | 23 | 11.2 | 72 | 35.1 | 50 | 24.4 | 28 | 13.7 | 32 | 15.6 | *x^2^* = 8.33 |
| Females | 19 | 8.4 | 93 | 41 | 69 | 30.4 | 16 | 7.1 | 30 | 13.2 | p=0.0803 |
| **Chronic Disease** |  |  |  |  |  |  |  |  |  |  | df = 4 |
| Males | 18 | 8.8 | 51 | 24.9 | 41 | 20 | 23 | 11.2 | 72 | 35.1 | *x^2^* = 3.02 |
| Females | 24 | 10.6 | 62 | 27.3 | 32 | 14.1 | 24 | 10.6 | 85 | 37.4 | p=0.5540 |
| **Public Health** |  |  |  |  |  |  |  |  |  |  | df = 4 |
| Males | 11 | 5.4 | 66 | 32.2 | 34 | 16.6 | 22 | 10.7 | 72 | 35.1 | *x^2^* = 3.80 |
| Females | 11 | 4.9 | 68 | 30 | 37 | 16.3 | 15 | 6.6 | 96 | 42.3 | p=0.4339 |
| **Tobacco** |  |  |  |  |  |  |  |  |  |  | df = 4 |
| Males | 16 | 7.8 | 69 | 33.7 | 50 | 24.4 | 36 | 17.6 | 34 | 16.6 | *x^2^* = 9.16 |
| Females | 11 | 4.9 | 99 | 43.6 | 61 | 26.9 | 23 | 10.1 | 33 | 14.5 | p=0.0573 |

**Supplemental Table 4. Hours of formal training in chronic disease prevention related-topics by degree type.**

|  | No Formal Training | | 1-5 hours | | 6-10 hours | | 11-15 hours | | >15 hours | | Chi-Square Test Results |
| --- | --- | --- | --- | --- | --- | --- | --- | --- | --- | --- | --- |
|  | Frequency | Percent | Frequency | Percent | Frequency | Percent | Frequency | Percent | Frequency | Percent |  |
| **Physical Activity** |  |  |  |  |  |  |  |  |  |  |  |
| MD | 107 | 38.9 | 118 | 42.9 | 35 | 12.7 | 6 | 2.2 | 9 | 3.3 | df = 8 |
| MD/MPH | 33 | 25.8 | 73 | 57 | 16 | 12.5 | 5 | 3.9 | 1 | 0.8 | *x^2^* = 18.89 |
| Other | 13 | 44.8 | 12 | 41.4 | 0 | 0 | 2 | 6.9 | 2 | 6.9 | p=0.0155 |
| **Nutrition** |  | |  | |  | |  | |  | |  |
| MD | 54 | 19.6 | 151 | 54.9 | 45 | 16.4 | 12 | 4.4 | 13 | 4.7 | df = 8 |
| MD/MPH | 14 | 10.9 | 78 | 60.9 | 28 | 21.9 | 6 | 4.7 | 2 | 1.6 | *x^2^* = 11.62 |
| Other | 5 | 17.2 | 14 | 48.3 | 5 | 17.2 | 2 | 6.9 | 3 | 10.3 | p=0.1689 |
| **Obesity** |  | |  | |  | |  | |  | |  |
| MD | 31 | 11.3 | 111 | 40.4 | 72 | 26.2 | 25 | 9.1 | 36 | 13.1 | df = 8 |
| MD/MPH | 9 | 7 | 37 | 28.9 | 42 | 32.8 | 18 | 14.1 | 22 | 17.2 | *x^2^* = 15.39 |
| Other | 2 | 6.9 | 17 | 58.6 | 5 | 17.2 | 1 | 3.5 | 4 | 13.8 | p=0.0519 |
| **Chronic Disease** |  | |  | |  | |  | |  | |  |
| MD | 36 | 13.1 | 91 | 33.1 | 55 | 20 | 31 | 11.3 | 62 | 22.6 | df = 8 |
| MD/MPH | 1 | 0.8 | 12 | 9.4 | 15 | 11.7 | 12 | 9.4 | 88 | 68.8 | *x^2^* = 92.48 |
| Other | 5 | 17.2 | 10 | 34.5 | 3 | 10.3 | 4 | 13.8 | 7 | 24.1 | p<0.0001 |
| **Public Health** |  | |  | |  | |  | |  | |  |
| MD | 20 | 7.3 | 121 | 44 | 61 | 22.2 | 36 | 13.1 | 37 | 13.5 | df = 8 |
| MD/MPH | 0 | 0 | 2 | 1.6 | 1 | 0.8 | 0 | 0 | 125 | 97.7 | *x^2^* = 269.15 |
| Other | 2 | 6.9 | 11 | 37.9 | 9 | 31 | 1 | 3.5 | 6 | 20.7 | p<0.0001 |
| **Tobacco** |  | |  | |  | |  | |  | |  |
| MD | 18 | 6.6 | 108 | 39.3 | 66 | 24 | 42 | 15.3 | 41 | 14.9 | df = 8 |
| MD/MPH | 8 | 6.3 | 47 | 36.7 | 38 | 29.7 | 13 | 10.2 | 22 | 17.2 | *x^2^* = 3.99 |
| Other | 1 | 3.5 | 13 | 44.8 | 7 | 24.1 | 4 | 13.8 | 4 | 13.8 | p=0.8584 |

**Supplemental Table 5. Hours of formal training in chronic disease prevention related-topics by medical specialty.**

|  | No Formal Training | | 1-5 hours | | 6-10 hours | | 11-15 hours | | >15 hours | | Chi-Square Test Results |
| --- | --- | --- | --- | --- | --- | --- | --- | --- | --- | --- | --- |
|  | Frequency | Percent | Frequency | Percent | Frequency | Percent | Frequency | Percent | Frequency | Percent |  |
| **Physical Activity** |  |  |  |  |  |  |  |  |  |  |  |
| Non-primary care | 91 | 35.1 | 119 | 46 | 30 | 11.6 | 8 | 3.1 | 11 | 4.3 | df = 8 |
| Primary care | 53 | 35.1 | 74 | 49 | 20 | 13.3 | 3 | 2 | 1 | 0.7 | *x^2^* = 9.95 |
| Unsure | 9 | 40.9 | 10 | 45.5 | 1 | 4.6 | 2 | 9.1 | 0 | 0 | p=0.2687 |
| **Nutrition** |  |  |  |  |  |  |  |  |  |  |  |
| Non-primary care | 40 | 15.4 | 145 | 56 | 47 | 18.2 | 12 | 4.6 | 15 | 5.8 | df = 8 |
| Primary care | 27 | 17.9 | 83 | 55 | 30 | 20 | 8 | 5.3 | 3 | 2 | *x^2^* = 10.36 |
| Unsure | 6 | 27.3 | 15 | 68.2 | 1 | 4.6 | 0 | 0 | 0 | 0 | p=0.2407 |
| **Obesity** |  |  |  |  |  |  |  |  |  |  |  |
| Non-primary care | 19 | 7.3 | 91 | 35.1 | 79 | 30.5 | 30 | 11.6 | 40 | 15.4 | df = 8 |
| Primary care | 16 | 10.6 | 65 | 43.1 | 35 | 23.2 | 14 | 9.3 | 21 | 13.9 | *x^2^* = 20.92 |
| Unsure | 7 | 31.8 | 9 | 40.9 | 5 | 22.7 | 0 | 0 | 1 | 4.6 | p=0.0074 |
| **Chronic Disease** |  |  |  |  |  |  |  |  |  |  |  |
| Non-primary care | 23 | 8.9 | 66 | 25.5 | 44 | 17 | 32 | 12.4 | 94 | 36.3 | df = 8 |
| Primary care | 12 | 8 | 39 | 25.8 | 26 | 17.2 | 14 | 9.3 | 60 | 39.7 | *x^2^* = 18.09 |
| Unsure | 7 | 31.8 | 8 | 36.4 | 3 | 13.6 | 1 | 4.6 | 3 | 13.6 | p=0.0206 |
| **Public Health** |  |  |  |  |  |  |  |  |  |  |  |
| Non-primary care | 13 | 5 | 83 | 32.1 | 45 | 17.4 | 28 | 10.8 | 90 | 34.8 | df = 8 |
| Primary care | 4 | 2.7 | 42 | 27.8 | 22 | 14.6 | 9 | 6 | 74 | 49 | *x^2^* = 29.14 |
| Unsure | 5 | 22.7 | 9 | 40.9 | 4 | 18.2 | 0 | 0 | 4 | 18.2 | p=0.0003 |
| **Tobacco** |  |  |  |  |  |  |  |  |  |  |  |
| Non-primary care | 11 | 4.3 | 1 | 35.1 | 71 | 27.4 | 42 | 16.2 | 44 | 17 | df = 8 |
| Primary care | 12 | 8 | 67 | 44.4 | 35 | 23.2 | 14 | 9.3 | 23 | 15.2 | *x^2^* = 17.62 |
| Unsure | 4 | 18.2 | 10 | 45.5 | 5 | 22.7 | 3 | 13.6 | 0 | 0 | p=0.0243 |

**Supplemental Table 6. Awareness of major chronic disease and diabetes prevention programs, overall.**

|  | Very Aware | | Somewhat Aware | | Neutral | | Somewhat Unaware | | Not at all Aware | |
| --- | --- | --- | --- | --- | --- | --- | --- | --- | --- | --- |
|  | Frequency | Percent | Frequency | Percent | Frequency | Percent | Frequency | Percent | Frequency | Percent |
| Chronic disease prevention programs | 48 | 11.1 | 159 | 36.8 | 65 | 15.1 | 99 | 22.9 | 61 | 14.1 |
| Diabetes prevention programs | 21 | 4.9 | 90 | 20.8 | 79 | 18.3 | 123 | 28.5 | 119 | 27.6 |

**Supplemental Table 7. Awareness of major chronic disease and diabetes prevention programs by year in medical school.**

|  | Very Aware | | Somewhat Aware | | Neutral | | Somewhat Unaware | | Not at all Aware | | Chi-Square Test Results |
| --- | --- | --- | --- | --- | --- | --- | --- | --- | --- | --- | --- |
|  | Frequency | Percent | Frequency | Percent | Frequency | Percent | Frequency | Percent | Frequency | Percent |  |
| **Chronic disease prevention programs** |  |  |  |  |  |  |  |  |  |  |  |
| 1st year | 13 | 10.2 | 39 | 30.7 | 16 | 12.6 | 35 | 27.6 | 24 | 18.9 | df = 12 |
| 2nd year | 10 | 9.1 | 41 | 37.3 | 17 | 15.5 | 24 | 21.8 | 18 | 16.4 | *x^2^* = 18.21 |
| 3rd year | 9 | 8.3 | 50 | 46.3 | 18 | 16.7 | 23 | 21.3 | 8 | 7.4 | p=0.1094 |
| 4th year | 16 | 18.4 | 29 | 33.3 | 14 | 16.1 | 17 | 19.5 | 11 | 12.6 |  |
| **Diabetes prevention programs** |  |  |  |  |  |  |  |  |  |  |  |
| 1st year | 3 | 2.4 | 18 | 14.2 | 20 | 15.8 | 43 | 33.9 | 43 | 33.9 | df = 12 |
| 2nd year | 3 | 2.7 | 28 | 25.5 | 21 | 19.1 | 23 | 20.9 | 35 | 31.8 | *x^2^* = 28.01 |
| 3rd year | 6 | 5.6 | 23 | 21.3 | 22 | 20.4 | 39 | 36.1 | 18 | 16.7 | p=0.0055 |
| 4th year | 9 | 10.3 | 21 | 24.1 | 16 | 18.4 | 18 | 20.7 | 23 | 26.4 |  |

**Supplemental Table 8. Awareness of major chronic disease and diabetes prevention programs by sex.**

|  | Very Aware | | Somewhat Aware | | Neutral | | Somewhat Unaware | | Not at all Aware | | Chi-Square Test Results |
| --- | --- | --- | --- | --- | --- | --- | --- | --- | --- | --- | --- |
|  | Frequency | Percent | Frequency | Percent | Frequency | Percent | Frequency | Percent | Frequency | Percent |  |
| **Chronic disease prevention programs** |  |  |  |  |  |  |  |  |  |  | df = 4 |
| Males | 28 | 13.7 | 67 | 32.7 | 32 | 15.6 | 46 | 22.4 | 32 | 15.6 | *x^2^* = 4.81 |
| Females | 20 | 8.8 | 92 | 40.5 | 33 | 14.5 | 53 | 23.4 | 29 | 12.8 | p=0.3069 |
| **Diabetes prevention programs** |  |  |  |  |  |  |  |  |  |  | df = 4 |
| Males | 13 | 6.3 | 35 | 17.1 | 36 | 17.6 | 58 | 28.3 | 63 | 30.7 | *x^2^* = 5.96 |
| Females | 8 | 3.5 | 55 | 24.2 | 43 | 18.9 | 65 | 28.6 | 56 | 24.7 | p=0.2021 |

**Supplemental Table 9. Awareness of major chronic disease and diabetes prevention programs by degree type.**

|  | Very Aware | | Somewhat Aware | | Neutral | | Somewhat Unaware | | Not at all Aware | | Chi-Square Test Results |
| --- | --- | --- | --- | --- | --- | --- | --- | --- | --- | --- | --- |
|  | Frequency | Percent | Frequency | Percent | Frequency | Percent | Frequency | Percent | Frequency | Percent |  |
| **Chronic disease prevention program** |  |  |  |  |  |  |  |  |  |  |  |
| MD | 14 | 5.1 | 85 | 30.9 | 44 | 16 | 81 | 29.5 | 51 | 18.6 | df = 8 |
| MD/MPH | 32 | 25 | 67 | 52.3 | 17 | 13.3 | 8 | 6.3 | 4 | 3.1 | *x^2^* = 82.37 |
| Other | 2 | 6.9 | 7 | 24.1 | 4 | 13.8 | 10 | 34.5 | 6 | 20.7 | p<.0001 |
| **Diabetes prevention programs** |  |  |  |  |  |  |  |  |  |  |  |
| MD | 8 | 2.9 | 37 | 13.5 | 44 | 16 | 91 | 33.1 | 95 | 34.6 | df = 8 |
| MD/MPH | 11 | 8.6 | 50 | 39.1 | 30 | 23.4 | 25 | 19.5 | 12 | 9.4 | *x^2^* = 65.96 |
| Other | 2 | 6.9 | 3 | 10.3 | 5 | 17.2 | 7 | 24.1 | 12 | 41.4 | p<.0001 |

**Supplemental Table 10. Awareness of major chronic disease and diabetes prevention programs by medical specialty.**

|  | Very Aware | | Somewhat Aware | | Neutral | | Somewhat Unaware | | Not at all Aware | | Chi-Square Test Results |
| --- | --- | --- | --- | --- | --- | --- | --- | --- | --- | --- | --- |
|  | Frequency | Percent | Frequency | Percent | Frequency | Percent | Frequency | Percent | Frequency | Percent |  |
| **Chronic disease prevention programs** |  |  |  |  |  |  |  |  |  |  |  |
| Non-primary care | 31 | 12 | 91 | 35.1 | 34 | 13.1 | 64 | 24.7 | 39 | 15.1 | df = 8 |
| Primary care | 16 | 10.6 | 61 | 40.4 | 28 | 18.5 | 28 | 18.5 | 18 | 11.9 | *x^2^* = 7.15 |
| Unsure | 1 | 4.6 | 7 | 31.8 | 3 | 13.6 | 7 | 31.8 | 4 | 18.2 | p=0.5210 |
| **Diabetes prevention programs** |  |  |  |  |  |  |  |  |  |  |  |
| Non-primary care | 15 | 5.8 | 47 | 18.2 | 50 | 19.3 | 75 | 29 | 72 | 27.8 | df = 8 |
| Primary care | 6 | 4 | 40 | 26.5 | 24 | 15.9 | 44 | 29.1 | 37 | 24.5 | *x^2^* = 10.33 |
| Unsure | 0 | 0 | 3 | 13.6 | 5 | 22.7 | 4 | 18.2 | 10 | 45.5 | p=0.2424 |

**Supplemental Table 11. Importance of formal training in chronic disease-related topics, overall.**

|  | Very Important | | Important | | Somewhat Important | | Not Very Important | | Not at all Important | |
| --- | --- | --- | --- | --- | --- | --- | --- | --- | --- | --- |
|  | Frequency | Percent | Frequency | Percent | Frequency | Percent | Frequency | Percent | Frequency | Percent |
| Physical Activity | 187 | 45.6 | 137 | 33.4 | 59 | 14.4 | 20 | 4.9 | 7 | 1.7 |
| Nutrition | 257 | 62.7 | 112 | 27.3 | 35 | 8.5 | 3 | 1 | 1 | 1.6 |
| Obesity | 265 | 64.6 | 104 | 25.4 | 34 | 8.3 | 4 | 1 | 4 | 1 |
| Chronic Disease | 271 | 66.1 | 108 | 26.3 | 26 | 6.3 | 2 | 0.5 | 3 | 0.7 |
| Public Health | 229 | 55.9 | 115 | 28.1 | 53 | 12.9 | 8 | 2 | 5 | 1.2 |
| Tobacco | 254 | 62 | 115 | 28.1 | 31 | 7.6 | 6 | 1.5 | 4 | 1 |

**Supplemental Table 12. Importance of formal training in chronic disease-related topics by year in medical school.**

|  | Very Important | | Important | | Somewhat Important | | Not Very Important | | Not at all Important | | Chi-Square Test Results |
| --- | --- | --- | --- | --- | --- | --- | --- | --- | --- | --- | --- |
|  | Frequency | Percent | Frequency | Percent | Frequency | Percent | Frequency | Percent | Frequency | Percent |  |
| **Physical Activity** |  |  |  |  |  |  |  |  |  |  |  |
| 1st year | 60 | 50 | 36 | 30 | 19 | 15.8 | 4 | 3.3 | 1 | 0.8 | df = 12 |
| 2nd year | 42 | 38.2 | 41 | 50.9 | 18 | 7.3 | 7 | 0 | 0 | 3.6 | *x^2^* = 20.22 |
| 3rd year | 53 | 54.1 | 29 | 29.6 | 12 | 12.2 | 1 | 1 | 3 | 3.1 | p=0.0630 |
| 4th year | 32 | 38.1 | 31 | 36.9 | 10 | 11.9 | 8 | 9.5 | 3 | 3.6 |  |
| **Nutrition** |  |  |  |  |  |  |  |  |  |  |  |
| 1st year | 75 | 62.5 | 35 | 29.2 | 9 | 7.5 | 1 | 0.8 | 0 | 0 | df = 12 |
| 2nd year | 60 | 55.6 | 35 | 32.4 | 12 | 11.1 | 1 | 0.9 | 0 | 0 | *x^2^* = 19.49 |
| 3rd year | 75 | 76.5 | 17 | 17.4 | 4 | 4.1 | 1 | 1 | 1 | 1 | p=0.0773 |
| 4th year | 47 | 56 | 25 | 29.8 | 10 | 11.9 | 0 | 0 | 2 | 2.4 |  |
| **Obesity** |  |  |  |  |  |  |  |  |  |  |  |
| 1st year | 76 | 63.3 | 30 | 25 | 12 | 10 | 2 | 1.7 | 0 | 0 | df = 12 |
| 2nd year | 69 | 63.9 | 27 | 25 | 11 | 10.2 | 1 | 1 | 0 | 0 | *x^2^* = 10.77 |
| 3rd year | 70 | 71.4 | 22 | 22.5 | 5 | 5.1 | 0 | 0 | 1 | 1 | p=0.5483 |
| 4th year | 50 | 59.5 | 25 | 29.8 | 6 | 7.1 | 1 | 1.2 | 2 | 2.4 |  |
| **Chronic Disease** |  |  |  |  |  |  |  |  |  |  |  |
| 1st year | 80 | 66.7 | 31 | 25.8 | 9 | 7.5 | 0 | 0 | 0 | 0 | df = 12 |
| 2nd year | 73 | 67.6 | 27 | 25 | 7 | 6.5 | 1 | 0.9 | 0 | 0 | *x^2^* = 7.83 |
| 3rd year | 64 | 65.3 | 26 | 26.5 | 6 | 6.1 | 1 | 1 | 1 | 1 | p=0.7985 |
| 4th year | 54 | 64.3 | 24 | 28.6 | 4 | 4.8 | 0 | 0 | 2 | 2.4 |  |
| **Public Health** |  |  |  |  |  |  |  |  |  |  |  |
| 1st year | 66 | 55 | 38 | 31.7 | 13 | 10.8 | 3 | 2.5 | 0 | 0 | df = 12 |
| 2nd year | 59 | 54.6 | 30 | 27.8 | 15 | 13.9 | 3 | 2.8 | 1 | 0.9 | *x^2^* = 8.75 |
| 3rd year | 58 | 59.2 | 26 | 26.5 | 12 | 12.2 | 1 | 1 | 1 | 1 | p=0.7241 |
| 4th year | 46 | 54.8 | 21 | 25 | 13 | 15.5 | 1 | 1.2 | 3 | 3.6 |  |
| **Tobacco** |  |  |  |  |  |  |  |  |  |  |  |
| 1st year | 71 | 59.2 | 37 | 30.8 | 10 | 8.3 | 2 | 1.7 | 0 | 0 | df = 12 |
| 2nd year | 68 | 63 | 28 | 25.9 | 9 | 8.3 | 3 | 2.8 | 0 | 0 | *x^2^* = 10.32 |
| 3rd year | 66 | 67.4 | 24 | 24.5 | 6 | 6.1 | 0 | 0 | 2 | 2 | p=0.5875 |
| 4th year | 49 | 58.3 | 26 | 31 | 6 | 7.1 | 1 | 1.2 | 2 | 2.4 |  |

**Supplemental Table 13. Importance of formal training in chronic disease-related topics by sex.**

|  | Very Important | | Important | | Somewhat Important | | Not Very Important | | Not at all Important | | Chi-Square Test Results |
| --- | --- | --- | --- | --- | --- | --- | --- | --- | --- | --- | --- |
|  | Frequency | Percent | Frequency | Percent | Frequency | Percent | Frequency | Percent | Frequency | Percent |  |
| **Physical Activity** |  |  |  |  |  |  |  |  |  |  | df = 4 |
| Males | 83 | 42.1 | 64 | 32.5 | 33 | 16.8 | 13 | 6.6 | 4 | 2 | *x^2^* = 5.11 |
| Females | 104 | 48.8 | 73 | 34.3 | 26 | 12.2 | 7 | 3.3 | 3 | 1.4 | p=0.2766 |
| **Nutrition** |  |  |  |  |  |  |  |  |  |  | df = 4 |
| Males | 111 | 56.4 | 60 | 30.5 | 21 | 10.7 | 3 | 1.5 | 2 | 1 | *x^2^* = 9.46 |
| Females | 146 | 68.5 | 52 | 24.4 | 14 | 6.6 | 0 | 0 | 1 | 0.5 | p=0.0505 |
| **Obesity** |  |  |  |  |  |  |  |  |  |  | df = 4 |
| Males | 115 | 58.4 | 57 | 28.9 | 19 | 9.6 | 4 | 2 | 2 | 1 | *x^2^* = 9.78 |
| Females | 150 | 70.4 | 47 | 22.1 | 15 | 7 | 0 | 0 | 1 | 0.5 | p=0.0443 |
| **Chronic Disease** |  |  |  |  |  |  |  |  |  |  | df = 4 |
| Males | 122 | 61.9 | 56 | 28.4 | 15 | 7.6 | 2 | 1 | 2 | 1 | *x^2^* = 5.17 |
| Females | 149 | 70 | 52 | 24.4 | 11 | 5.2 | 0 | 0 | 1 | 0.5 | p=0.2703 |
| **Public Health** |  |  |  |  |  |  |  |  |  |  | df = 4 |
| Males | 91 | 46.2 | 65 | 33 | 34 | 17.3 | 3 | 1.5 | 4 | 2 | *x^2^* = 17.55 |
| Females | 138 | 64.8 | 50 | 23.5 | 19 | 8.9 | 5 | 2.4 | 1 | 0.5 | p=0.0015 |
| **Tobacco** |  |  |  |  |  |  |  |  |  |  | df = 4 |
| Males | 113 | 57.4 | 58 | 29.4 | 19 | 9.6 | 5 | 2.5 | 2 | 1 | *x^2^* = 6.73 |
| Females | 141 | 66.2 | 57 | 26.8 | 12 | 5.6 | 1 | 0.5 | 2 | 0.9 | p=0.1510 |

**Supplemental Table 14. Importance of formal training in chronic disease-related topics by degree type.**

|  | Very Important | | Important | | Somewhat Important | | Not Very Important | | Not at all Important | | Chi-Square Test Results |
| --- | --- | --- | --- | --- | --- | --- | --- | --- | --- | --- | --- |
|  | Frequency | Percent | Frequency | Percent | Frequency | Percent | Frequency | Percent | Frequency | Percent |  |
| **Physical Activity** |  |  |  |  |  |  |  |  |  |  |  |
| MD | 115 | 43.9 | 89 | 34 | 37 | 14.1 | 16 | 6.1 | 5 | 1.9 | df = 8 |
| MD/MPH | 61 | 50.8 | 39 | 32.5 | 17 | 14.2 | 2 | 1.7 | 1 | 0.8 | *x^2^* = 6.26 |
| Other | 11 | 39.3 | 9 | 32.1 | 5 | 17.9 | 2 | 7.1 | 1 | 3.6 | p=0.6178 |
| **Nutrition** |  |  |  |  |  |  |  |  |  |  |  |
| MD | 161 | 61.5 | 72 | 27.5 | 24 | 9.2 | 3 | 1.2 | 2 | 0.8 | df = 8 |
| MD/MPH | 80 | 66.7 | 32 | 26.7 | 8 | 6.7 | 0 | 0 | 0 | 0 | *x^2^* = 6.97 |
| Other | 16 | 57.1 | 8 | 28.6 | 3 | 10.7 | 0 | 0 | 1 | 3.6 | p=0.5398 |
| **Obesity** |  |  |  |  |  |  |  |  |  |  |  |
| MD | 159 | 60.7 | 72 | 27.5 | 26 | 9.9 | 3 | 1.2 | 2 | 0.8 | df = 8 |
| MD/MPH | 88 | 73.3 | 26 | 21.7 | 6 | 5 | 0 | 0 | 0 | 0 | *x^2^* = 12.92 |
| Other | 18 | 64.3 | 6 | 21.4 | 2 | 7.1 | 1 | 3.6 | 1 | 3.6 | p=0.1146 |
| **Chronic Disease** |  |  |  |  |  |  |  |  |  |  |  |
| MD | 166 | 63.4 | 75 | 28.6 | 17 | 6.5 | 2 | 0.8 | 2 | 0.8 | df = 8 |
| MD/MPH | 86 | 71.7 | 28 | 23.3 | 6 | 5 | 0 | 0 | 0 | 0 | *x^2^* = 8.86 |
| Other | 19 | 67.9 | 5 | 17.9 | 3 | 10.7 | 0 | 0 | 1 | 3.6 | p=0.3542 |
| **Public Health** |  |  |  |  |  |  |  |  |  |  |  |
| MD | 124 | 47.3 | 84 | 32.1 | 44 | 16.8 | 6 | 2.3 | 4 | 1.5 | df = 8 |
| MD/MPH | 91 | 75.8 | 23 | 19.2 | 5 | 4.2 | 1 | 0.8 | 0 | 0 | *x^2^* = 31.47 |
| Other | 14 | 50 | 8 | 28.6 | 4 | 14.3 | 1 | 3.6 | 1 | 3.6 | p=0.0001 |
| **Tobacco** |  |  |  |  |  |  |  |  |  |  |  |
| MD | 155 | 59.2 | 80 | 30.5 | 20 | 7.6 | 5 | 1.9 | 2 | 0.8 | df = 8 |
| MD/MPH | 81 | 67.5 | 29 | 24.2 | 9 | 7.5 | 0 | 0 | 1 | 0.8 | *x^2^* = 7.66 |
| Other | 18 | 64.3 | 6 | 21.4 | 2 | 7.1 | 1 | 3.6 | 1 | 3.6 | p=0.4674 |

**Supplemental Table 15. Importance of formal training in chronic disease-related topics by medical specialty.**

|  | Very Important | | Important | | Somewhat Important | | Not Very Important | | Not at all Important | | Chi-Square Test Results |
| --- | --- | --- | --- | --- | --- | --- | --- | --- | --- | --- | --- |
|  | Frequency | Percent | Frequency | Percent | Frequency | Percent | Frequency | Percent | Frequency | Percent |  |
| **Physical Activity** |  |  |  |  |  |  |  |  |  |  |  |
| Non-primary care | 104 | 42.5 | 84 | 34.3 | 36 | 14.7 | 15 | 6.1 | 6 | 2.5 | df = 8 |
| Primary care | 72 | 50.4 | 46 | 32.2 | 21 | 14.7 | 3 | 2.1 | 1 | 0.7 | *x^2^* = 7.79 |
| Unsure | 11 | 50 | 7 | 31.8 | 2 | 9.1 | 2 | 9.1 | 0 | 0 | p=0.4546 |
| **Nutrition** |  |  |  |  |  |  |  |  |  |  |  |
| Non-primary care | 144 | 58.7 | 73 | 29.8 | 23 | 9.4 | 3 | 1.2 | 2 | 0.8 | df = 8 |
| Primary care | 99 | 69.2 | 32 | 22.4 | 11 | 7.7 | 0 | 0 | 1 | 0.7 | *x^2^* = 6.51 |
| Unsure | 14 | 63.6 | 7 | 31.8 | 1 | 4.6 | 0 | 0 | 0 | 0 | p=0.5898 |
| **Obesity** |  |  |  |  |  |  |  |  |  |  |  |
| Non-primary care | 153 | 62.5 | 64 | 26.1 | 23 | 9.4 | 3 | 1.2 | 2 | 0.8 | df = 8 |
| Primary care | 98 | 68.5 | 34 | 23.8 | 10 | 7 | 0 | 0 | 1 | 0.7 | *x^2^* = 6.38 |
| Unsure | 14 | 63.6 | 6 | 27.3 | 1 | 4.6 | 1 | 4.6 | 0 | 0 | p=0.6048 |
| **Chronic Disease** |  |  |  |  |  |  |  |  |  |  |  |
| Non-primary care | 152 | 62 | 71 | 29 | 18 | 7.4 | 2 | 0.8 | 2 | 0.8 | df = 8 |
| Primary care | 105 | 73.4 | 30 | 21 | 7 | 4.9 | 0 | 0 | 1 | 0.7 | *x^2^* = 6.76 |
| Unsure | 14 | 63.6 | 7 | 31.8 | 1 | 4.6 | 0 | 0 | 0 | 0 | p=0.5628 |
| **Public Health** |  |  |  |  |  |  |  |  |  |  |  |
| Non-primary care | 121 | 49.4 | 75 | 30.6 | 38 | 15.5 | 7 | 2.9 | 4 | 1.6 | df = 8 |
| Primary care | 97 | 67.8 | 32 | 22.4 | 13 | 9.1 | 0 | 0 | 1 | 0.7 | *x^2^* = 17.05 |
| Unsure | 11 | 50 | 8 | 36.4 | 2 | 9.1 | 1 | 4.6 | 0 | 0 | p=0.0296 |
| **Tobacco** |  |  |  |  |  |  |  |  |  |  |  |
| Non-primary care | 146 | 59.6 | 72 | 29.4 | 19 | 7.8 | 5 | 2 | 3 | 1.2 | df = 8 |
| Primary care | 95 | 66.4 | 36 | 25.2 | 11 | 7.7 | 0 | 0 | 1 | 0.7 | *x^2^* = 6.24 |
| Unsure | 13 | 59.1 | 7 | 31.8 | 1 | 4.6 | 1 | 4.6 | 0 | 0 | p=0.6200 |

**Supplemental Table 16. Importance of applied experiences in chronic disease prevention, overall.**

|  | Very Important | | Important | | Somewhat Important | | Not Very Important | | Not at all Important | |
| --- | --- | --- | --- | --- | --- | --- | --- | --- | --- | --- |
|  | Frequency | Percent | Frequency | Percent | Frequency | Percent | Frequency | Percent | Frequency | Percent |
| Tobacco | 105 | 25.7 | 211 | 51.6 | 63 | 15.4 | 21 | 5.1 | 9 | 2.2 |
| Alcohol | 106 | 25.9 | 217 | 52.9 | 62 | 15.1 | 17 | 4.2 | 8 | 2 |
| Obesity | 133 | 32.4 | 217 | 52.9 | 39 | 9.5 | 12 | 2.9 | 9 | 2.2 |
| Chronic Disease | 156 | 38.1 | 204 | 49.9 | 32 | 7.8 | 10 | 2.4 | 7 | 1.7 |

**Supplemental Table 17. Importance of applied experiences in chronic disease prevention by year in medical school.**

|  | Very Important | | Important | | Somewhat Important | | Not Very Important | | Not at all Important | | Chi-Square Test Results |
| --- | --- | --- | --- | --- | --- | --- | --- | --- | --- | --- | --- |
|  | Frequency | Percent | Frequency | Percent | Frequency | Percent | Frequency | Percent | Frequency | Percent |  |
| **Smoking** |  |  |  |  |  |  |  |  |  |  |  |
| 1st year | 30 | 25.2 | 65 | 54.6 | 16 | 13.5 | 7 | 5.9 | 1 | 0.8 |  |
| 2nd year | 29 | 26.9 | 59 | 54.6 | 10 | 9.3 | 8 | 7.4 | 2 | 1.9 | df = 12 |
| 3rd year | 28 | 28.6 | 48 | 49 | 17 | 17.4 | 3 | 3.1 | 2 | 2 | *x^2^* = 15.00 |
| 4th year | 18 | 21.4 | 39 | 46.4 | 20 | 23.8 | 3 | 3.6 | 4 | 4.8 | p=0.2416 |
| **Alcohol** |  |  |  |  |  |  |  |  |  |  |  |
| 1st year | 31 | 25.8 | 66 | 55 | 17 | 14.2 | 5 | 4.2 | 1 | 0.8 |  |
| 2nd year | 28 | 25.9 | 61 | 56.5 | 10 | 9.3 | 7 | 6.5 | 2 | 1.9 | df = 12 |
| 3rd year | 26 | 26.5 | 49 | 50 | 18 | 18.4 | 3 | 3.1 | 2 | 2 | *x^2^* = 9.73 |
| 4th year | 21 | 25 | 41 | 48.8 | 17 | 20.2 | 2 | 2.4 | 3 | 3.6 | p=0.6400 |
| **Obesity** |  |  |  |  |  |  |  |  |  |  |  |
| 1st year | 43 | 35.8 | 63 | 52.5 | 10 | 8.3 | 2 | 1.7 | 2 | 1.7 |  |
| 2nd year | 38 | 35.2 | 55 | 50.9 | 6 | 5.6 | 7 | 6.5 | 2 | 1.9 | df = 12 |
| 3rd year | 30 | 30.6 | 53 | 54.1 | 12 | 12.2 | 1 | 1 | 2 | 2 | *x^2^* = 13.37 |
| 4th year | 22 | 26.2 | 46 | 54.8 | 11 | 13.1 | 2 | 2.4 | 3 | 3.6 | p=0.3427 |
| **Chronic disease** |  |  |  |  |  |  |  |  |  |  |  |
| 1st year | 47 | 39.2 | 65 | 54.2 | 6 | 5 | 2 | 1.7 | 0 | 0 |  |
| 2nd year | 46 | 42.6 | 47 | 43.5 | 6 | 5.6 | 7 | 6.5 | 2 | 1.9 | df = 12 |
| 3rd year | 35 | 35.7 | 48 | 49 | 13 | 13.3 | 0 | 0 | 2 | 2 | *x^2^* = 22.64 |
| 4th year | 28 | 33.7 | 44 | 53 | 7 | 8.4 | 1 | 1.2 | 3 | 3.6 | p=0.0309 |

**Supplemental Table 18. Importance of applied experiences in chronic disease prevention by sex.**

|  | Very Important | | Important | | Somewhat Important | | Not Very Important | | Not at all Important | | Chi-Square Test Results |
| --- | --- | --- | --- | --- | --- | --- | --- | --- | --- | --- | --- |
|  | Frequency | Percent | Frequency | Percent | Frequency | Percent | Frequency | Percent | Frequency | Percent |  |
| **Smoking** |  |  |  |  |  |  |  |  |  |  | df = 4 |
| Males | 50 | 25.4 | 100 | 50.8 | 28 | 14.2 | 11 | 5.6 | 8 | 4.1 | *x^2^* = 6.54 |
| Females | 55 | 25.9 | 111 | 52.4 | 35 | 16.5 | 10 | 4.7 | 1 | 0.5 | p=0.1623 |
| **Alcohol** |  |  |  |  |  |  |  |  |  |  | df = 4 |
| Males | 53 | 26.9 | 101 | 51.3 | 27 | 13.7 | 9 | 4.6 | 7 | 3.6 | *x^2^* = 6.01 |
| Females | 53 | 24.9 | 116 | 54.5 | 35 | 16.4 | 8 | 3.8 | 1 | 0.5 | p=0.1982 |
| **Obesity** |  |  |  |  |  |  |  |  |  |  | df = 4 |
| Males | 60 | 30.5 | 102 | 51.8 | 21 | 10.7 | 6 | 3.1 | 8 | 4.1 | *x^2^* = 7.11 |
| Females | 73 | 34.3 | 115 | 54 | 18 | 8.5 | 6 | 2.8 | 1 | 0.5 | p=0.1301 |
| **Chronic disease** |  |  |  |  |  |  |  |  |  |  | df = 4 |
| Males | 69 | 35.2 | 99 | 50.5 | 18 | 9.2 | 4 | 2 | 6 | 3.1 | *x^2^* = 6.03 |
| Females | 87 | 40.9 | 105 | 49.3 | 14 | 6.6 | 6 | 2.8 | 1 | 0.5 | p=0.1970 |

**Supplemental Table 19. Importance of applied experiences in chronic disease prevention by degree type.**

|  | Very Important | | Important | | Somewhat Important | | Not Very Important | | Not at all Important | | Chi-Square Test Results |
| --- | --- | --- | --- | --- | --- | --- | --- | --- | --- | --- | --- |
|  | Frequency | Percent | Frequency | Percent | Frequency | Percent | Frequency | Percent | Frequency | Percent |  |
| **Smoking** |  |  |  |  |  |  |  |  |  |  |  |
| MD | 62 | 23.8 | 136 | 52.1 | 39 | 14.9 | 17 | 6.5 | 7 | 2.7 | df = 8 |
| MD/MPH | 35 | 29.2 | 62 | 51.7 | 21 | 17.5 | 1 | 0.8 | 1 | 0.8 | *x^2^* = 10.49 |
| Other | 8 | 28.6 | 13 | 46.4 | 3 | 10.7 | 3 | 10.7 | 1 | 3.6 | p=0.2320 |
| **Alcohol** |  |  |  |  |  |  |  |  |  |  |  |
| MD | 63 | 24.1 | 141 | 53.8 | 38 | 14.5 | 12 | 5 | 7 | 2.7 | df = 8 |
| MD/MPH | 35 | 29.2 | 66 | 55 | 18 | 15 | 1 | 0.8 | 0 | 0 | *x^2^* = 13.35 |
| Other | 8 | 28.6 | 10 | 35.7 | 6 | 21.4 | 3 | 10.7 | 1 | 3.6 | p=0.1002 |
| **Obesity** |  |  |  |  |  |  |  |  |  |  |  |
| MD | 75 | 28.6 | 145 | 55.3 | 25 | 9.5 | 10 | 3.8 | 7 | 2.7 | df = 8 |
| MD/MPH | 50 | 41.7 | 59 | 49.2 | 10 | 8.3 | 1 | 0.8 | 0 | 0 | *x^2^* = 14.71 |
| Other | 8 | 28.6 | 13 | 46.4 | 4 | 14.3 | 1 | 3.6 | 2 | 7.1 | p=0.0651 |
| **Chronic disease** |  |  |  |  |  |  |  |  |  |  |  |
| MD | 90 | 34.5 | 137 | 52.5 | 20 | 7.7 | 8 | 3.1 | 6 | 2.3 | df = 8 |
| MD/MPH | 57 | 47.5 | 54 | 45 | 8 | 6.7 | 1 | 0.8 | 0 | 0 | *x^2^* = 11.62 |
| Other | 9 | 32.1 | 13 | 46.4 | 4 | 14.3 | 1 | 3.6 | 1 | 3.6 | p=0.1691 |

**Supplemental Table 20. Importance of applied experiences in chronic disease prevention by medical specialty.**

|  | Very Important | | Important | | Somewhat Important | | Not Very Important | | Not at all Important | | Chi-Square Test Results |
| --- | --- | --- | --- | --- | --- | --- | --- | --- | --- | --- | --- |
|  | Frequency | Percent | Frequency | Percent | Frequency | Percent | Frequency | Percent | Frequency | Percent |  |
| **Smoking** |  |  |  |  |  |  |  |  |  |  |  |
| Non-primary care | 59 | 24.2 | 128 | 52.5 | 38 | 15.6 | 11 | 4.5 | 8 | 3.3 | df = 8 |
| Primary care | 39 | 27.3 | 75 | 52.5 | 19 | 13.3 | 9 | 6.3 | 1 | 0.7 | *x^2^* = 7.97 |
| Unsure | 7 | 31.8 | 8 | 36.4 | 6 | 27.3 | 1 | 4.6 | 0 | 0 | p=0.4365 |
| **Alcohol** |  |  |  |  |  |  |  |  |  |  |  |
| Non-primary care | 61 | 24.9 | 132 | 53.9 | 36 | 14.7 | 9 | 3.7 | 7 | 2.9 | df = 8 |
| Primary care | 37 | 25.9 | 76 | 53.2 | 22 | 15.4 | 7 | 4.9 | 1 | 0.7 | *x^2^* = 4.78 |
| Unsure | 8 | 36.4 | 9 | 40.9 | 4 | 18.2 | 1 | 4.6 | 0 | 0 | p=0.7803 |
| **Obesity** |  |  |  |  |  |  |  |  |  |  |  |
| Non-primary care | 68 | 27.8 | 138 | 56.3 | 24 | 9.8 | 8 | 3.3 | 7 | 2.9 | df = 8 |
| Primary care | 59 | 41.3 | 67 | 46.9 | 12 | 8.4 | 4 | 2.8 | 1 | 0.7 | *x^2^* = 10.66 |
| Unsure | 6 | 27.3 | 12 | 54.6 | 3 | 13.6 | 0 | 0 | 1 | 4.6 | p=0.2217 |
| **Chronic disease** |  |  |  |  |  |  |  |  |  |  |  |
| Non-primary care | 84 | 34.4 | 128 | 52.5 | 20 | 8.2 | 6 | 2.5 | 6 | 2.5 | df = 8 |
| Primary care | 64 | 44.8 | 63 | 44.1 | 11 | 7.7 | 4 | 2.8 | 1 | 0.7 | *x^2^* = 7.20 |
| Unsure | 8 | 36.4 | 13 | 59.1 | 1 | 4.6 | 0 | 0 | 0 | 0 | p=0.5150 |

**Supplemental Table 21. Best time to receive formal training in chronic disease prevention, overall.**

|  | 1st Year | | 2nd Year | | 3rd Year | | Residency | | Internship/Fellowship | | Other | | Never | |
| --- | --- | --- | --- | --- | --- | --- | --- | --- | --- | --- | --- | --- | --- | --- |
|  | Frequency | Percent | Frequency | Percent | Frequency | Percent | Frequency | Percent | Frequency | Percent | Frequency | Percent | Frequency | Percent |
| **Chronic disease prevention training** | 199 | 48.5 | 105 | 25.6 | 67 | 16.3 | 12 | 2.9 | 0 | 0 | 22 | 5.4 | 5 | 1.2 |

**Supplemental Table 22. Best time to receive formal training in chronic disease prevention by year in medical school.**

|  | 1st Year | | 2nd Year | | 3rd Year | | Residency | | Internship/Fellowship | | Other | | Never | | Chi-Square Test Results |
| --- | --- | --- | --- | --- | --- | --- | --- | --- | --- | --- | --- | --- | --- | --- | --- |
|  | Frequency | Percent | Frequency | Percent | Frequency | Percent | Frequency | Percent | Frequency | Percent | Frequency | Percent | Frequency | Percent |  |
| **Year in medical school**  1st year | 78 | 65 | 15 | 12.5 | 16 | 13.3 | 4 | 3.3 | 0 | 0 | 5 | 4.2 | 2 | 1.7 | df = 15 |
| 2nd year | 56 | 51.9 | 22 | 20.4 | 17 | 15.7 | 5 | 4.6 | 0 | 0 | 7 | 6.5 | 1 | 0.9 | *x^2^* = 43.29 |
| 3rd year | 34 | 34.7 | 34 | 34.7 | 21 | 21.4 | 1 | 1 | 0 | 0 | 8 | 8.2 | 0 | 0 | p=0.0001 |
| 4th year | 31 | 36.9 | 34 | 40.5 | 13 | 15.5 | 2 | 2.4 | 0 | 0 | 2 | 2.4 | 2 | 2.4 |  |

**Supplemental Table 23. Best time to receive formal training in chronic disease prevention by sex.**

|  | 1st Year | | 2nd Year | | 3rd Year | | Residency | | Internship/Fellowship | | Other | | Never | | Chi-Square Test Results |
| --- | --- | --- | --- | --- | --- | --- | --- | --- | --- | --- | --- | --- | --- | --- | --- |
|  | Frequency | Percent | Frequency | Percent | Frequency | Percent | Frequency | Percent | Frequency | Percent | Frequency | Percent | Frequency | Percent |  |
| **Gender** |  |  |  |  |  |  |  |  |  |  |  |  |  |  | df = 5 |
| Males | 91 | 46.2 | 48 | 24.4 | 34 | 17.3 | 9 | 4.6 | 0 | 0 | 10 | 5.1 | 5 | 2.5 | *x^2^* = 9.81 |
| Females | 108 | 50.7 | 57 | 26.8 | 33 | 15.5 | 3 | 1.4 | 0 | 0 | 12 | 5.6 | 0 | 0 | p=0.0808 |

**Supplemental Table 24. Best time to receive formal training in chronic disease prevention by degree type.**

|  | 1st Year | | 2nd Year | | 3rd Year | | Residency | | Internship/Fellowship | | Other | | Never | | Chi-Square Test Results |
| --- | --- | --- | --- | --- | --- | --- | --- | --- | --- | --- | --- | --- | --- | --- | --- |
|  | Frequency | Percent | Frequency | Percent | Frequency | Percent | Frequency | Percent | Frequency | Percent | Frequency | Percent | Frequency | Percent |  |
| **Degree** |  |  |  |  |  |  |  |  |  |  |  |  |  |  |  |
| MD | 122 | 46.6 | 78 | 29.8 | 43 | 16.4 | 5 | 1.9 | 0 | 0 | 10 | 3.8 | 4 | 1.5 | df = 10 |
| MD/MPH | 65 | 54.2 | 20 | 16.7 | 20 | 16.7 | 4 | 3.3 | 0 | 0 | 11 | 9.2 | 0 | 0 | *x^2^* = 21.09 |
| Other | 12 | 42.9 | 7 | 25 | 4 | 14.3 | 3 | 10.7 | 0 | 0 | 1 | 3.6 | 1 | 3.6 | p=0.0205 |

**Supplemental Table 25. Best time to receive formal training in chronic disease prevention by medical specialty.**

|  | 1st Year | | 2nd Year | | 3rd Year | | Residency | | Internship/Fellowship | | Other | | Never | | Chi-Square Test Results |
| --- | --- | --- | --- | --- | --- | --- | --- | --- | --- | --- | --- | --- | --- | --- | --- |
|  | Frequency | Percent | Frequency | Percent | Frequency | Percent | Frequency | Percent | Frequency | Percent | Frequency | Percent | Frequency | Percent |  |
| **Specialty** |  |  |  |  |  |  |  |  |  |  |  |  |  |  |  |
| Non-Primary   Care | 125 | 51 | 57 | 23.3 | 39 | 15.9 | 7 | 2.9 | 0 | 0 | 13 | 5.3 | 4 | 1.6 | df = 10 |
| Primary Care | 65 | 45.5 | 43 | 30.1 | 24 | 16.8 | 4 | 2.8 | 0 | 0 | 7 | 4.9 | 0 | 0 | *x^2^* = 7.57 |
| Unsure | 9 | 40.9 | 5 | 22.7 | 4 | 18.2 | 1 | 4.6 | 0 | 0 | 2 | 9.1 | 1 | 4.6 | p=0.6704 |

**Supplemental Table 26. Best time to receive applied experiences in chronic disease prevention, overall.**

|  | 1st Year | | 2nd Year | | 3rd Year | | Residency | | Internship/Fellowship | | Other | | Never | |
| --- | --- | --- | --- | --- | --- | --- | --- | --- | --- | --- | --- | --- | --- | --- |
|  | Frequency | Percent | Frequency | Percent | Frequency | Percent | Frequency | Percent | Frequency | Percent | Frequency | Percent | Frequency | Percent |
| **Chronic disease prevention training** | 178 | 43.4% | 151 | 36.8% | 173 | 42.2% | 99 | 24.1% | 58 | 14.1% | 20 | 4.9% | 178 | 43.4% |

* Since respondents were able to select more than one response for this survey item, significance testing could not be conducted.

**Supplemental Table 27. Best time to receive applied experiences in chronic disease prevention by year in medical school.**

|  | 1st Year | | 2nd Year | | 3rd Year | | Residency | | Internship/Fellowship | | Other | | Never | |
| --- | --- | --- | --- | --- | --- | --- | --- | --- | --- | --- | --- | --- | --- | --- |
|  | Frequency | Percent | Frequency | Percent | Frequency | Percent | Frequency | Percent | Frequency | Percent | Frequency | Percent | Frequency | Percent |
| **Year in medical school**  1st year | 71 | 59.2 | 42 | 35.0 | 36 | 30.0 | 21 | 17.5 | 16 | 13.3 | 7 | 5.8 | 71 | 59.2 |
| 2nd year | 51 | 47.2 | 35 | 32.4 | 47 | 43.5 | 31 | 28.7 | 24 | 22.2 | 9 | 8.3 | 51 | 47.2 |
| 3rd year | 32 | 32.7 | 40 | 40.8 | 52 | 53.1 | 24 | 24.5 | 8 | 8.2 | 2 | 2.0 | 32 | 32.7 |
| 4th year | 24 | 28.6 | 34 | 40.5 | 38 | 45.2 | 23 | 27.4 | 10 | 11.9 | 2 | 2.4 | 24 | 28.6 |

* Since respondents were able to select more than one response for this survey item, significance testing could not be conducted.

**Supplemental Table 28. Best time to receive applied experiences in chronic disease prevention by sex.**

|  | 1st Year | | 2nd Year | | 3rd Year | | Residency | | Internship/Fellowship | | Other | | Never | |
| --- | --- | --- | --- | --- | --- | --- | --- | --- | --- | --- | --- | --- | --- | --- |
|  | Frequency | Percent | Frequency | Percent | Frequency | Percent | Frequency | Percent | Frequency | Percent | Frequency | Percent | Frequency | Percent |
| **Gender** |  |  |  |  |  |  |  |  |  |  |  |  |  |  |
| Males | 77 | 39.1 | 66 | 33.5 | 83 | 42.1 | 50 | 25.4 | 34 | 17.3 | 13 | 6.6 | 77 | 39.1 |
| Females | 101 | 47.4 | 85 | 39.9 | 90 | 42.3 | 49 | 23.0 | 24 | 11.3 | 7 | 3.3 | 101 | 47.4 |

* Since respondents were able to select more than one response for this survey item, significance testing could not be conducted.

**Supplemental Table 29. Best time to receive applied experiences in chronic disease prevention by degree type.**

|  | 1st Year | | 2nd Year | | 3rd Year | | Residency | | Internship/Fellowship | | Other | | Never | |
| --- | --- | --- | --- | --- | --- | --- | --- | --- | --- | --- | --- | --- | --- | --- |
|  | Frequency | Percent | Frequency | Percent | Frequency | Percent | Frequency | Percent | Frequency | Percent | Frequency | Percent | Frequency | Percent |
| **Degree** |  |  |  |  |  |  |  |  |  |  |  |  |  |  |
| MD | 108 | 41.2 | 97 | 37.0 | 105 | 40.1 | 56 | 21.4 | 34 | 13.0 | 9 | 3.4 | 108 | 41.2 |
| MD/MPH | 59 | 49.2 | 45 | 37.5 | 56 | 46.7 | 37 | 30.8 | 19 | 15.8 | 9 | 7.5 | 59 | 49.2 |
| Other | 11 | 39.3 | 9 | 32.1 | 12 | 42.9 | 6 | 21.4 | 5 | 17.9 | 2 | 7.1 | 11 | 39.3 |

* Since respondents were able to select more than one response for this survey item, significance testing could not be conducted.

**Supplemental Table 30. Best time to receive applied experiences in chronic disease prevention by medical specialty.**

|  | 1st Year | | 2nd Year | | 3rd Year | | Residency | | Internship/Fellowship | | Other | | Never | |
| --- | --- | --- | --- | --- | --- | --- | --- | --- | --- | --- | --- | --- | --- | --- |
|  | Frequency | Percent | Frequency | Percent | Frequency | Percent | Frequency | Percent | Frequency | Percent | Frequency | Percent | Frequency | Percent |
| **Specialty** |  |  |  |  |  |  |  |  |  |  |  |  |  |  |
| Non-Primary   Care | 105 | 42.9 | 81 | 33.1 | 102 | 41.6 | 50 | 20.4 | 29 | 11.8 | 9 | 3.7 | 105 | 42.9 |
| Primary Care | 63 | 44.1 | 58 | 40.6 | 65 | 45.5 | 45 | 31.5 | 24 | 16.8 | 9 | 6.3 | 63 | 44.1 |
| Unsure | 10 | 45.5 | 12 | 54.5 | 6 | 27.3 | 4 | 18.2 | 5 | 22.7 | 2 | 9.1 | 10 | 45.5 |

* Since respondents were able to select more than one response for this survey item, significance testing could not be conducted.
